# HLF promotes ovarian cancer progression and chemoresistance via regulating Hippo signaling pathway

**DOI:** 10.1038/s41419-023-06076-5

**Published:** 2023-09-14

**Authors:** Tao Han, Tingsong Chen, Lujun Chen, Kerui Li, Daimin Xiang, Lei Dou, Hengyu Li, Yubei Gu

**Affiliations:** 1 Department of Oncology, The First Affiliated Hospital of China Medical University, Shenyang, 110001 Liaoning China; 2 Department of Cancer Intervention, Seventh People’s Hospital of Shanghai University of TCM, Shanghai, 200001 China; 3 Postgraduate College, China Medical University, Shenyang, 110001 China; 4 Clinical Cancer Institute, Center for Translational Medicine, Naval Military Medical University, Shanghai, 200433 China; 5 Department of hepatobiliary surgery, East Hospital, School of Medicine, Tongji University, Shanghai, 200120 China; 6 Department of Gynecology, The First Affiliated Hospital of China Medical University, Shenyang, 110001 Liaoning China; 7 Department of Breast and Thyroid Surgery, Changhai Hospital, Naval Military Medical University, Shanghai, 200433 China; 8 Department of Gastroenterology, Ruijin Hospital Affiliated to Shanghai Jiao Tong University School of Medicine, Shanghai, China

**Keywords:** Oncogenes, Cancer stem cells, Ovarian cancer, Predictive markers, Tumour biomarkers

## Abstract

Hepatic leukemia factor (HLF) is aberrantly expressed in human malignancies. However, the role of HLF in the regulation of ovarian cancer (OC) remains unknown. Herein, we reported that HLF expression was upregulated in OC tissues and ovarian cancer stem cells (CSCs). Functional studies have revealed that HLF regulates OC cell stemness, proliferation, and metastasis. Mechanistically, HLF transcriptionally activated Yes-associated protein 1 (YAP1) expression and subsequently modulated the Hippo signaling pathway. Moreover, we found that miR-520e directly targeted HLF 3′-UTR in OC cells. miR-520e expression was negatively correlated with HLF and YAP1 expression in OC tissues. The combined immunohistochemical (IHC) panels exhibited a better prognostic value for OC patients than any of these components alone. Importantly, the HLF/YAP1 axis determines the response of OC cells to carboplatin treatment and HLF depletion or the YAP1 inhibitor verteporfin abrogated carboplatin resistance. Analysis of patient-derived xenografts (PDXs) further suggested that HLF might predict carboplatin benefits in OC patients. In conclusion, these findings suggest a crucial role of the miR-520e/HLF/YAP1 axis in OC progression and chemoresistance, suggesting potential therapeutic targets for OC.

## Introduction

Ovarian cancer (OC) is a common and deadly malignancy worldwide [1]. Owing to the absence of obvious early symptoms, most OC patients are diagnosed at an advanced stage. Surgical resection, followed by platinum-based chemotherapy, is the current standard treatment for OC [2]. However, most patients develop resistance to platinum-based chemotherapy and relapse over time [3]. Chemotherapy resistance is a prognostic factor for poor prognosis and has been proven to be associated with the presence of cancer stem cells (CSCs) [4]. Therefore, it is crucial to urgently investigate the mechanisms of therapy resistance and identify novel therapeutic targets.

Hepatic leukemia factor (HLF) is a member of the proline and acidic amino acid-rich basic leucine zipper (PAR bZIP) transcription factor family, which includes two other members: albumin D-site-binding protein (DBP) and thyrotroph embryonic factor (TEF) [5]. The PAR bZIP transcription factor family is closely associated with the circadian clock in cells. Simultaneous deletion of the PAR bZIP transcription factor family (DBP, TEF, and HLF) shortens the lifespan of mice with symptoms of cardiac hypertrophy, hypotension, hypoaldosteronism, and epilepsy [6, 7]. HLF participates in the regulation of hematopoietic stem cells and hematopoietic malignancies [8–10]. Moreover, our previous study showed that HLF plays a vital role in the regulation of liver fibrosis [11]. Dysregulation of HLF has also been observed in many cancer types, including liver cancer [12], breast cancer [13], intrahepatic cholangiocarcinoma [14], glioma [15], and lung adenocarcinoma [16]. Nevertheless, the role of HLF in OC progression and chemoresistance remains obscure.

Yes-associated protein 1 (YAP1), a transcriptional coactivator of Hippo signaling, plays a pivotal role in tumor proliferation, metastasis, and chemoresistance [17, 18]. Location 11q22 in the human YAP1 locus is amplified in various human cancers and cancer cell lines [19]. In addition, YAP1 overexpression occurs in a large proportion of human cancers, including ovarian, gastric, liver, and prostate [20–23]. Elevated YAP1 expression closely correlates with reduced survival in human cancers [24, 25]. YAP1 may play a role in the progression of OC [25, 26]; however, the exact mechanism underlying YAP1 activation in OC remains vague.

In this study, we discovered that HLF drives OC progression and chemoresistance by transactivating YAP1, suggesting that HLF is a novel prognostic biomarker and a potential target for OC therapy.

## Results

### HLF upregulation predicts poor prognosis in OC patients

**Fig. 1:**
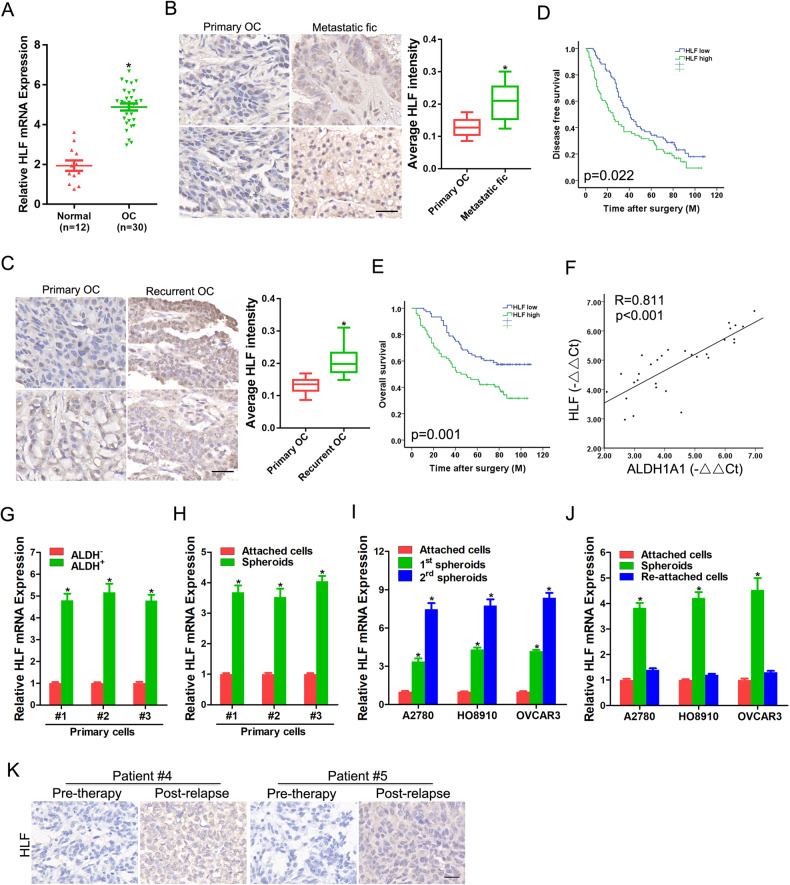
Expression of HLF is elevated in ovarian cancer tissues and cancer stem cells. **A** Real-time PCR analysis of HLF expression in human OC tissues (*n* = 30) and normal tissues (*n* = 12). **B** Representative images of IHC staining of HLF in matched human OC (*n* = 16) and distal metastatic tissues (*n* = 16). Scale bar = 25 μm. **C** Representative images of IHC staining of HLF in matched human OC (*n* = 16) and relapsing OC tissues (*n* = 16). Scale bar = 25 μm. **D** The disease-free survival time after surgery in OC patients was compared between the “HLF low, *n* = 76” and “HLF high, *n* = 76” groups. **E** The overall survival time after surgery in OC patients was compared between the “HLF low, *n* = 76” and “HLF high, *n* = 76” groups. **F** The correlation between the level of HLF and ALDH1A1 in OC tissues (*n* = 30) was determined by real-time PCR analysis. **G** Real-time PCR analysis of HLF expression in flow-sorted ALDH^+^ and ALDH^−^ primary OC cells. **H** Real-time PCR analysis of HLF expression in primary OC adherent cells and spheres. **I** Real-time PCR analysis of HLF expression in serial passages of OC cell spheroids. **J** Real-time PCR analysis of HLF expression HLF expression in OC adherent cells, spheres, and reattached cells. **K** Representative images of IHC staining of HLF in matched human OC taken before carboplatin therapy (pre-therapy) and after carboplatin resistance (post-relapse). Scale bar = 25 μm.

To explore the pathological role of HLF in OC, human OC tissues were analyzed to determine HLF expression levels. Notably, HLF transcript levels were significantly higher in OC tissues than in normal ovarian tissues (Fig. 1A). Strikingly, we observed that HLF expression was further elevated in distal metastatic tissues compared to the corresponding OC tissues, indicating the potential role of HLF in OC metastasis (Fig. 1B). Moreover, the levels of HLF were markedly upregulated in recurrent OC compared to those in primary OC (Fig. 1C).

HLF expression was determined in 152 OC tissues by immunohistochemistry using tissue microarrays (TMA). Interestingly, HLF overexpression was associated with aggressive OC features (Supplementary Table S2). Importantly, HLF overexpression was significantly associated with disease-free survival (DFS) and overall survival (OS) in the univariate analysis (Fig. 1D, E). Multivariate analysis revealed that a high HLF level was an independent predictor of poor prognosis in OC patients (Supplementary Tables S3 and 4), which further indicates that HLF may be a potential prognostic biomarker in OC.

### HLF was upregulated in ovarian CSCs

Aldehyde dehydrogenase (ALDH) is a well-known ovarian CSC marker [27]. Interestingly, HLF levels were positively correlated with the expression of ALDH1A1 in OC tissues (Fig. 1F). Moreover, the expression level of HLF was significantly upregulated in flow-sorted ALDH^+^ primary OC cells compared with ALDH^-^ primary OC cells (Fig. 1G). Compared to adherent cells, HLF expression was increased in OC spheres derived from human primary OC cells (Fig. 1H). We also observed that HLF expression was upregulated in flow-sorted ALDH^+^ OC cells and enriched OC cell spheroids (Supplementary Fig. S1A–D). During serial passages of OC cell spheroids, HLF expression gradually increased (Fig. 1I and Supplementary Fig. S1E). Notably, the HLF level was reduced to its original level when the spheres were reattached (Fig. 1J and Supplementary Fig. S1F). Previous studies have reported that ovarian CSCs are closely associated with tumor progression and chemoresistance [28]. Consistently, high HLF expression was observed in clinically relapsed tumors after carboplatin therapy (Fig. 1K). Taken together, these results suggest that HLF is upregulated in ovarian CSCs.

### HLF regulates stemness, proliferation, and metastasis in OC cells

**Fig. 2:**
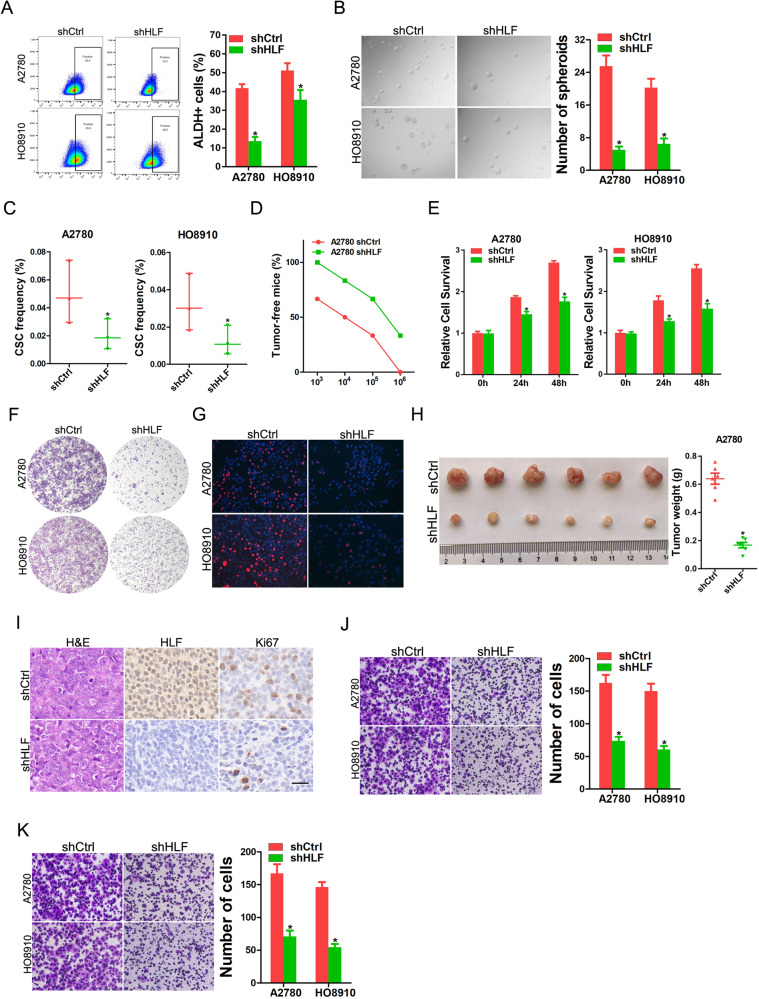
HLF regulates OC cell stemness, proliferation, and metastasis. **A** Flow cytometry analysis of ALDH^+^ populations in shHLF and control OC cells. **B** Representative images of spheroids generated from shHLF and control OC cells. The number of spheroids was counted and compared. **C** The frequency of CSCs in shHLF and control OC cells was compared by in vitro limiting dilution assay. **D** In vivo limiting dilution assay of HLF knockdown and control OC cells. Tumors were observed over 2 months; *n* = 6 for each group. **E** Proliferation of shHLF and control OC cells was evaluated by Cell Counting Kit 8 Assay. **F** shHLF or control OC cells were subjected to the colony formation assay. The formed colonies were fixed and stained with crystal violet; representative images are presented. **G** Representative images of EdU staining of proliferating shHLF or control OC cells. EdU^+^ cells were stained with red immunofluorescence. The nuclei were counterstained with DAPI. Scale bar = 50 μm. **H**. In total, 2 × 10^6^ A2780 shHLF or control cells were subcutaneously inoculated into nude mice (*n* = 6) and excised 6 weeks later. The weight of xenografted tumors from different groups was compared. **I** Representative images of IHC staining of Ki67 and HLF in xenografted tumors. Scale bar = 25 μm. **J** Migration assay was performed using polycarbonate membrane inserts in a 24-well plate. **K**. The invasive properties of shHLF or control OC cells were analyzed using a Matrigel-coated Boyden chamber.

To investigate the function of HLF in OC progression, we generated HLF-knockdown and -overexpressing OC cell lines by stably infecting them with a lentivirus (Supplementary Fig. S2A, B). HLF knockdown decreased the ALDH^+^ cell population and impaired the spheroid formation of OC cells (Fig. 2A, B). Consistently, overexpression of HLF increased the ALDH^+^ cell population and enhanced the spheroid-forming ability of OC cells (Supplementary Fig. S2C, D). In addition, in vitro limiting dilution assays revealed that the proportion of CSCs was decreased in HLF-depleted OC cells and increased in HLF-overexpressing OC cells (Fig. 2C and Supplementary Fig. S2E). In vivo limiting dilution assay revealed that the suppression of HLF significantly reduced tumor incidence (Fig. 2D).

Cell Counting Kit-8 assays, 5-ethynyl-20-deoxyuridine (EdU) staining, and colony formation assays consistently showed that HLF knockdown suppressed OC cell growth and survival (Fig. 2E–G). Consistently, proliferation and colony formation of OC cells was promoted by ectopic HLF expression (Supplementary Fig. S2F–H). Furthermore, HLF-depleted OC cells formed xenografts with decreased size in mice (Fig. 2H, I). Next, we investigated whether HLF regulates the migration and invasion of OC cells. As expected, HLF inhibition significantly decreased the migration and invasion abilities of OC cells (Fig. 2J, K). Increased HLF expression consistently enhanced the migration and invasion of OC cells (Supplementary Fig. S2I, J). Collectively, these results demonstrated that HLF plays a critical role in OC progression.

### HLF activates YAP1 through transcription

**Fig. 3:**
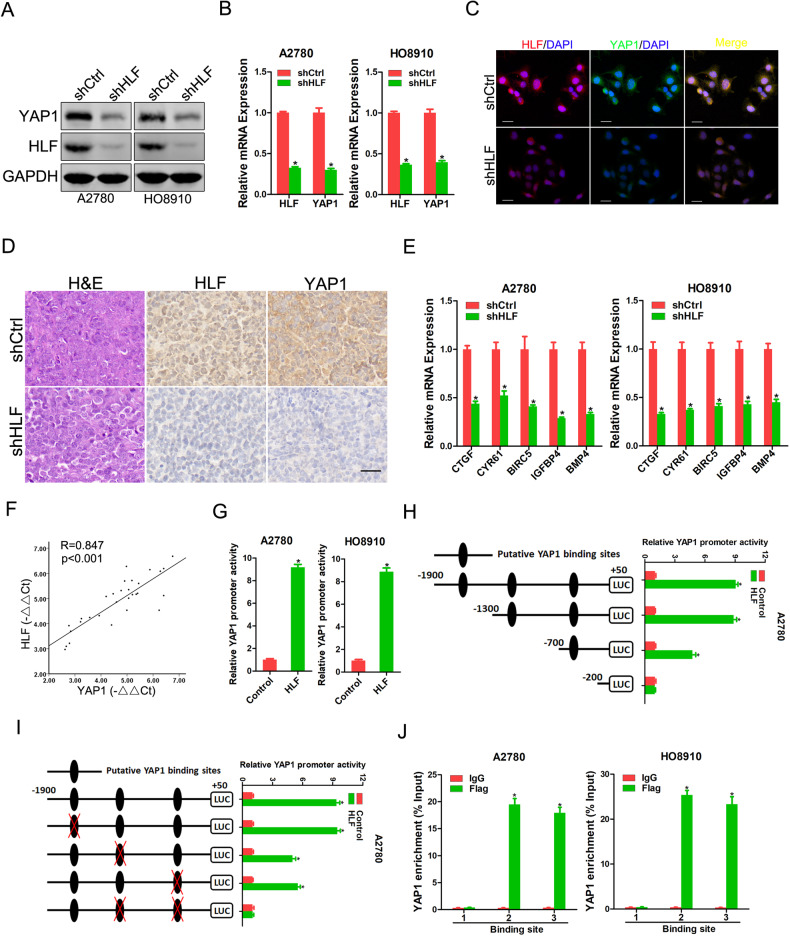
HLF transcriptionally activates YAP1 in OC cells. **A** Western blot analysis of the protein expression of HLF and YAP1 in shHLF or control OC cells. **B** Real-time PCR analysis of the mRNA expression of HLF and YAP1 in shHLF or control OC cells. **C** Representative images of dual immunofluorescence staining of HLF and YAP1 in A2780 shHLF or control cells. The nuclei were counterstained with DAPI. Scale bar = 20 μm. **D** Representative images of H&E and IHC staining of HLF and YAP1 in xenografted tumors formed by A2780 shHLF or control cells. Scale bar = 25 μm. **E** Real-time PCR analysis of the mRNA expression of YAP1 targeted genes in shHLF or control OC cells. **F** The correlation between the level of HLF and YAP1 in OC tissues (*n* = 30) was determined using real-time PCR analysis. **G** The luciferase reporter activity of the YAP1 promoter was measured in HLF overexpression or control OC cells. **H** Deletion analysis identified HLF-responsive regions in the YAP1 promoter. Serially truncated YAP1 promoter constructs were transfected into A2780 HLF overexpressing and control cells, and relative luciferase activities were determined. **I** Selective mutation analysis identified HLF-responsive regions in the YAP1 promoter. Serially mutated YAP1 promoter constructs were transfected into A2780 HLF overexpressing and control cells, and relative luciferase activities were determined. **J** OC cells were subjected to ChIP assay with anti-Flag or anti-IgG antibody followed by real-time PCR.

Several signaling pathways, including TGF-β/SMAD, PI3-K/Akt, JAK/STAT3, and Hippo, play critical roles in the progression of various cancers [29–32]. Herein, our data showed that TGF-β/SMAD, PI3-K/Akt, or JAK/STAT3 pathway was not influenced by HLF knockdown (Supplementary Fig. S3A). However, YAP1, a transcriptional co-activator in Hippo signaling, was decreased by HLF depletion and increased by HLF overexpression in OC cells (Fig. 3A and Supplementary Fig. S3B). Real-time PCR analysis confirmed that YAP1 activation was impaired in HLF-depleted OC cells, whereas the opposite effect was observed when HLF was overexpressed (Fig. 3B and Supplementary Fig. S3C). Immunofluorescence staining further confirmed the consistent downregulation of YAP1 in HLF-knockdown OC cells (Fig. 3C and Supplementary Fig. S3D). A decreased level of YAP1 expression was detected in xenografts formed by HLF-depleted OC cells compared to that in xenografts formed by control cells (Fig. 3D). Moreover, YAP1 target gene expression was decreased in HLF-depleted OC cells, whereas the opposite effect was observed when HLF was overexpressed (Fig. 3E and Supplementary Fig. S3E). Notably, a correlation between HLF expression and the levels of YAP1 was observed in human OC tissues using real-time PCR (Fig. 3F). Furthermore, we found that the activation of the YAP1 promoter could be enhanced via the ectopic expression of HLF (Fig. 3G). Sequence analysis revealed three putative HLF binding sites in the YAP1 promoter (Supplementary Fig. S3F). Serial deletion and site-directed mutagenesis revealed that the second and third HLF-binding sites were critical for HLF-induced YAP1 transactivation (Fig. 3H, I). Significant enrichment of HLF in the promoter region of YAP1 was detected using ChIP-qPCR (Fig. 3J). Taken together, these results suggest that HLF transactivates YAP1 expression in OC cells.

### YAP1 is responsible for HLF-mediated OC progression

**Fig. 4:**
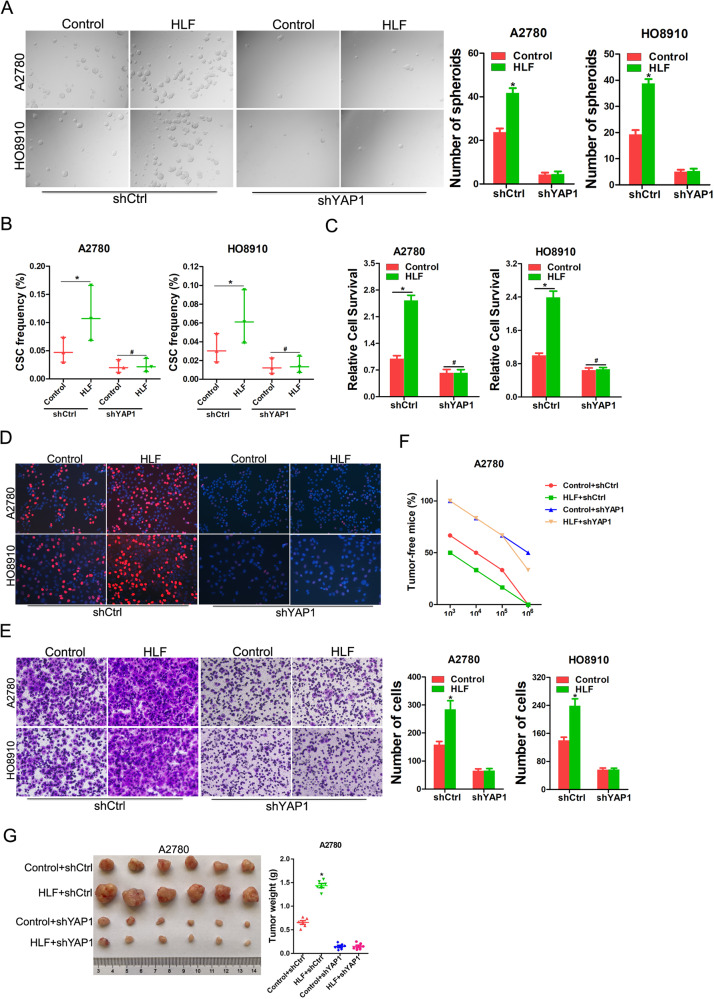
HLF activates YAP1 to promote OC progression. **A** A2780/HO8910 HLF and control cells infected with YAP1 knockdown or control virus were subjected to spheroids formation. **B** A2780/HO8910 HLF and control cells infected with YAP1 knockdown or control virus were subjected to in vitro limiting dilution assay. **C** A2780/HO8910 HLF and control cells infected with YAP1 knockdown or control virus were subjected to CCK-8 analysis. **D** A2780/HO8910 HLF and control cells infected with YAP1 knockdown or control virus were subjected to EdU staining. **E** A2780/HO8910 HLF and control cells infected with YAP1 knockdown or control virus were subjected to Matrigel invasion chamber assay. **F** A2780 HLF and control cells infected with YAP1 knockdown or control virus were subjected to in vivo limiting dilution assay. Tumors were observed over 2 months; *n* = 6 for each group. **G** Representative xenograft images at 8 weeks after the injection of indicated OC cells into nude mice. The weight of xenografts in each group (*n* = 6) was calculated.

We further explored whether OC progression is promoted by HLF via YAP1. Notably, the self-renewal, proliferation, and invasive capacities of HLF-knockdown OC cells were restored by the introduction of YAP1 (Supplementary Fig. S4A–E). Consistently, the knockdown of YAP1 abolished HLF overexpression and enhanced the self-renewal, proliferation, and invasive abilities of OC cells (Fig. 4A–E). Moreover, YAP1 depletion abrogated HLF-enhanced tumorigenesis and malignant proliferation of OC cells in vivo (Fig. 4F, G). Taken together, the upregulation of YAP1 partially contributed to the functional effects of HLF on OC cells.

### miR-520e suppresses OC progression via targeting HLF/YAP1 signaling

**Fig. 5:**
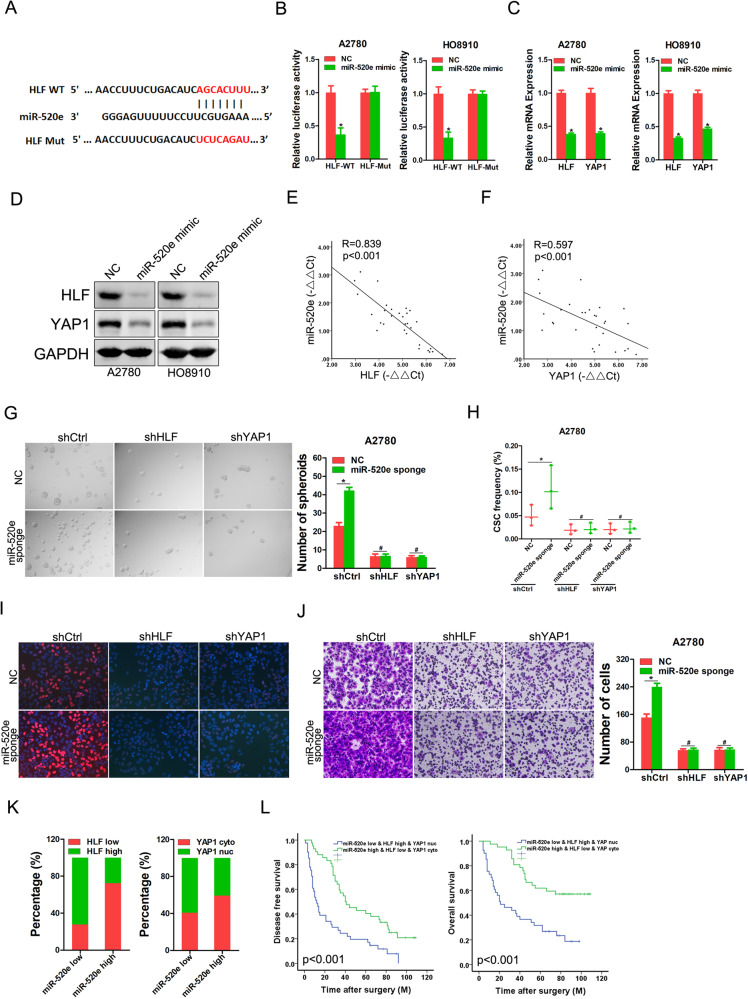
miR-520e inhibits OC progression via targeting HLF/YAP1. **A** A potential target site for miR-520e in the 3′UTR of human HLF mRNA, as predicted by the program TargetScan. To disrupt the interaction between miR-52e and HLF mRNA, the target site was mutated. **B** Luciferase reporter assays performed in miR-520e overexpression and control OC cells transfected with wild-type or mutant HLF 3′UTR constructs. **C** Real-time PCR analysis mRNA expression of HLF and YAP1 in miR-520e overexpression and control OC cells. **D** Western blot analysis protein expression of HLF and YAP1 in miR-520e overexpression and control OC cells. **E** The correlation between the level of miR-520e and HLF in human OC cells (*n* = 30) was determined using real-time PCR analysis. **F** The correlation between the level of miR-520e and YAP1 in human OC cells (*n* = 30) was determined using real-time PCR analysis. **G** A2780 miR-520e sponge and control cells infected with HLF or YAP1 knockdown virus were subjected to spheroids formation. **H** A2780 miR-520e sponge and control cells infected with HLF or YAP1 knockdown virus were subjected to in vitro limiting dilution assay. **I** A2780 miR-520e sponge and control cells infected with HLF or YAP1 knockdown virus were subjected to EdU staining. **J** A2780 miR-520e sponge and control cells infected with HLF or YAP1 knockdown virus were subjected to Matrigel invasion chamber assay. **K** The correlation between miR-520e levels, HLF expression, and nuclear YAP1 in OC tissues from cohort 1 was evaluated using a chi-square test. **L** Kaplan–Meier analysis of disease-free survival and overall survival in OC patients (*n* = 89) according to the expression of miR-520e-HLF-YAP1 axis.

To investigate the upstream regulatory mechanism of HLF in OC, OC cells were treated with a DNA methylation or histone deacetylase inhibitor. As shown in Supplementary Fig. S5A, B, our data suggest that DNA methylation or histone acetylation had no impact on HLF expression. Interestingly, we found that inhibition of Dicer, an enzyme controlling the microRNA (miRNA) processing, significantly increased HLF expression (Supplementary Fig. S5C), suggesting that these miRNAs may be involved in the upregulation of HLF expression in OC cells. Next, we searched the TargetScan database and identified 91 conserved candidate miRNAs (Supplementary Fig. S5D, Table S5), among which miR-520e was chosen for further study. To confirm the binding between miR-520e and HLF 3′-UTR, we performed assays with wild-type or mutated HLF 3′-UTR-coupled luciferase reporters (Fig. 5A). Luciferase activity was suppressed by miR-520e overexpression and enhanced via miR-520e knockdown in OC cells transfected with wild-type HLF 3′-UTR, mutation of which abrogated these effects (Fig. 5B and Supplementary Fig. S5E). Real-time PCR and western blot analyses revealed that the expression of HLF and its downstream gene YAP1 decreased in miR-520e overexpression OC cells and increased in miR-520e knockdown in OC cells (Fig. 5C, D, and Supplementary Fig. S5F, G). Consistent with this finding, the expression levels of HLF, YAP1, and miR-520e negatively correlated in human OC cells (Fig. 5E, F).

We evaluated the effects of miR-520e on various malignant properties of human OC cell lines in vitro. Overexpression of miR-520e significantly inhibited self-renewal, proliferation, colony formation, migration, and invasion of OC cells in vitro (Supplementary Fig. S5H, M). Next, we investigated whether miR-520e suppressed OC progression by regulating HLF/YAP1 expression. As expected, the knockdown of HLF or YAP1 abrogated the self-renewal ability, ovarian CSCs frequency, proliferation, and metastatic capacity of miR-520e-depleted OC cells (Fig. 5G–J and Supplementary Fig. S6A–E). Taken together, these data indicate that miR-520e inhibits OC progression by targeting the HLF/YAP1 signaling pathway.

We further tested the correlation between these molecules in OC patients and found a negative correlation between miR-520e, HLF, and the nuclear accumulation of YAP1 expression (Fig. 5K). Importantly, patients with low miR-520e levels, high HLF levels, and nuclear accumulation of YAP1 exhibited significantly poorer survival (Fig. 5L). Therefore, our patient data demonstrate that the miR-520e-HLF-YAP1 axis is activated in OC and correlates with worse clinical outcomes.

### HLF/YAP1 axis determines the response of carboplatin in OC

**Fig. 6:**
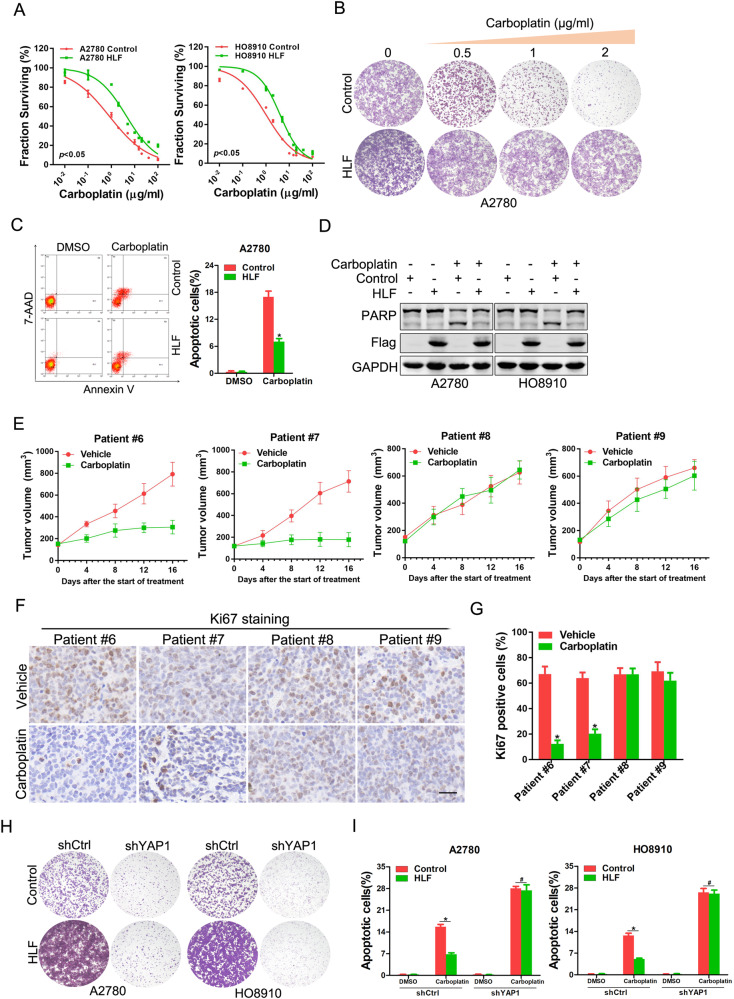
HLF determines the response of carboplatin in OC. **A** A2780/HO8910 HLF and control cells were treated with carboplatin for 48 h, and their cell survival curves were calculated. **B** A2780 HLF and control cells were treated with carboplatin for 10 days, and their colony growth was examined. **C** A2780 HLF and control cells were treated with carboplatin (4 μg/ml) for 48 h, and their apoptosis was examined using flow cytometry. **D** A2780/HO8910 HLF and control cells were treated with carboplatin (4 μg/ml) for 48 h and then subjected to western blot assay. **E** PDXs derived from the primary OCs with low HLF levels (Patients #6 and 7) or high HLF levels (Patients #8 and 9) were treated with carboplatin (60 mg/kg) or saline for 15 days (*n* = 5 for each group). The xenograft growth was monitored. **F** PDXs derived from indicated patients treated with saline or carboplatin were subjected to Ki67 staining. Representative views are presented. Scale bar = 25 μm. **G** PDXs derived from indicated patients treated with saline or carboplatin were subjected to Ki67 staining. The proportion of the Ki67 positive cells was quantified. **H** A2780/HO8910 HLF and control cells were infected with the shYAP1 virus. The cells were then treated with carboplatin in a 12-well dish for 10 days. **I** A2780/HO8910 HLF and control cells were infected with the shYAP1 virus. The cells were then treated with carboplatin (4 μg/ml) for 48 h, and their apoptosis was examined using flow cytometry.

Considering the crucial role of CSCs in the aggravation of chemoresistance, we investigated the effects of HLF on OC cell sensitivity to carboplatin. Notably, HLF-overexpressing OC cells were more resistant to carboplatin than control cells, as shown by an increase in IC_50_ (Fig. 6A). Additionally, HLF overexpression significantly decreased the carboplatin-induced inhibition of OC cell growth and apoptosis (Fig. 6B–D and Supplementary Fig. S7A, B). Next, patient-derived xenografts (PDXs) were established using fresh patient OC tissues to validate the correlation between HLF levels and the carboplatin response (Supplementary Fig. S7C). We found that PDXs derived from tumors with high HLF levels were resistant to carboplatin treatment (Fig. 6E). Moreover, decreased expression of Ki67, a marker of proliferating cells, was only detected in PDXs treated with carboplatin if they were derived from tumors with low HLF levels (Fig. 6F, G). Importantly, YAP1 inhibition reduced carboplatin resistance in HLF-overexpressing OC cells (Fig. 6H, I). Together, these results demonstrate that the HLF/YAP1 axis determines the response of OC to carboplatin.

### Targeting HLF/YAP1 axis restores carboplatin response in OC

**Fig. 7:**
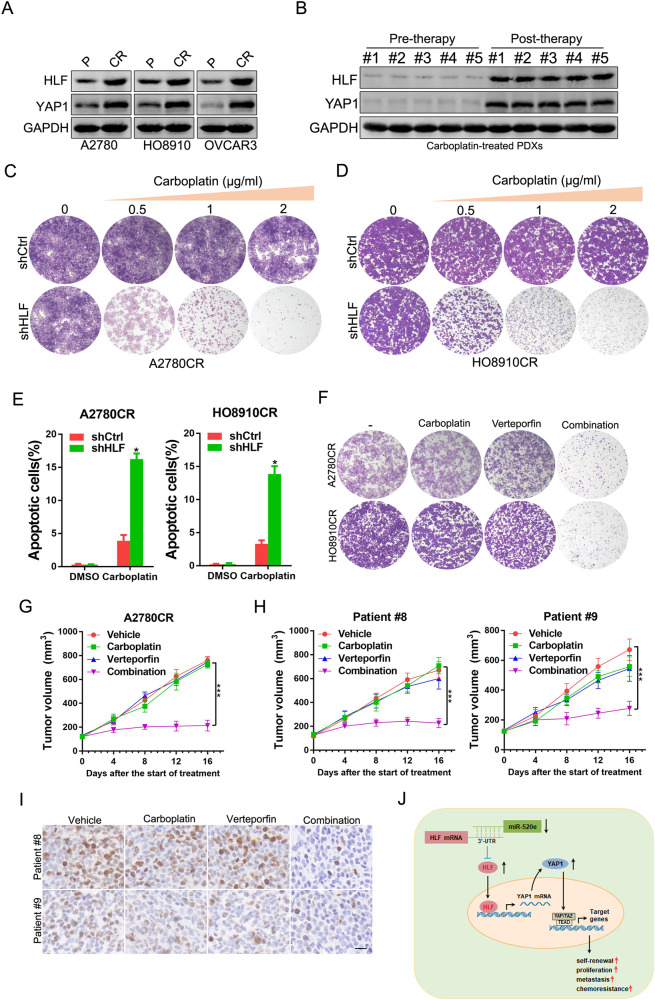
Targeting HLF/YAP1 axis restores carboplatin response in OC. **A** Western blot analysis of indicated proteins in A2780/HO8910 and A2780CR/HO8910CR cells. **B** Western blot analysis of indicated proteins in carboplatin pre-therapy and post-therapy PDXs. **C** A2780CR shHLF and control cells were treated with carboplatin for 10 days, and their colony growth was examined. **D** HO8910CR shHLF and control cells were treated with carboplatin for 10 days, and their colony growth was examined. **E** A2780CR/HO8910CR shHLF and control cells were treated with carboplatin (4 μg/ml) for 48 h, and their apoptosis was examined using flow cytometry. **F** A2780CR/HO8910CR cells were treated with carboplatin or/and verteporfin for 10 days, and their colony growth was examined. **G** Tumor growth curves of mice after the subcutaneous injection of A2780CR cells and intraperitoneal administration of carboplatin (60 mg/kg, once every other day) or/and verteporfin (100 mg/kg, twice a week), *n* = 5 for each group. The xenograft growth was monitored. Data are presented as the mean ± SEM. **H** PDXs derived from the primary OC patients (Patients #8 and 9) treated with carboplatin (60 mg/kg, once every other day) or/and verteporfin (100 mg/kg, once every other day). The xenograft growth was monitored. Data are presented as the mean ± SEM. **I** Schematic model of the mechanism underlying HLF-driven OC progression and chemoresistance.

Notably, the expression of HLF and YAP1 was increased in carboplatin-resistant OC cell lines (Fig. 7A). Consistently, high expression levels of HLF and YAP1 were observed in carboplatin-resistant PDXs (Fig. 7B). Importantly, we found that interference of HLF sensitized carboplatin-resistant OC cells to carboplatin-induced growth inhibition and apoptosis (Fig. 7C–E). Next, the YAP inhibitor, verteporfin, was investigated in vitro and in vivo. The combination of verteporfin and carboplatin resulted in notable growth suppression in carboplatin-resistant OC cells, whereas neither treatment alone inhibited growth (Fig. 7F, G). Moreover, verteporfin re-sensitized the PDXs to carboplatin treatment (Fig. 7H), accompanied by decreased levels of ki67 in post-treatment tumors (Fig. 7I). In addition, no notable weight loss was observed in mice during treatment (Supplementary Fig. S8A). Taken together, these data suggest that the HLF/YAP1 axis may serve as a potential therapeutic target for overcoming carboplatin resistance in OC.

## Discussion

Although the diagnosis and treatment of OC have improved, morbidity and mortality rates continue to increase. Therefore, the mechanisms underlying OC tumorigenesis and drug resistance require further investigation. In the present study, we reported that the downregulation of miR-520e results in the upregulation of HLF in OC tissues and ovarian CSCs. We found that HLF promoted OC stemness, proliferation, and metastasis by transactivating YAP1. Moreover, the HLF/YAP1 axis promoted carboplatin resistance in OC cells (Fig. 7J). These findings suggest that HLF is a potential prognostic biomarker and therapeutic target in OC.

Limited prior studies have indicated that HLF can act either as an oncogene or as a tumor suppressor, depending on the context. Our previous studies reported that HLF transactivates c-Jun to enhance the tumor-initiating cell (TICs)-like properties of hepatoma cells, thus promoting HCC progression and sorafenib resistance [12]. We have also shown that HLF regulates ferroptosis, development, and chemoresistance in triple-negative breast cancer by activating tumor macrophage crosstalk [13]. Conversely, HLF acts as a tumor suppressor in glioma and lung adenocarcinoma [15, 16]. Herein, we demonstrated that HLF levels were increased in OC tissues and ovarian CSCs. Functional experiments showed that HLF promotes OC stemness, proliferation, and metastasis both in vitro and in vivo, suggesting that HLF plays multiple roles in OC progression.

Previous studies have shown that YAP1 plays essential roles in OC cell proliferation and metastasis and controls chemotherapy escape [33]. In addition, YAP1 is involved in the regulation of TICs or CSCs [34]. YAP1 promotes tissue growth and cell viability by regulating the activity of multiple transcription factors, including CTGF, CYR61, BIRC5, IGFBP4, and BMP4 [35]. However, the mechanism underlying YAP1 upregulation is not well understood. In the present study, based on bioinformatics analysis, YAP1 was identified as a potential target with a promoter region containing an HLF-binding site. Subsequently, our study confirmed that HLF transcriptionally upregulated YAP1 expression to facilitate CSC properties, proliferation, and metastasis in OC cells. Considering the important role of the HLF/YAP1 axis in OC progression, targeting this axis may be a novel therapeutic strategy for OC.

Increasing evidence indicates that miRNA expression is dysregulated in OC. Both loss and gain of miRNA function contribute to OC progression and chemoresistance [36, 37]. We hypothesized that the loss or suppression of miRNAs targeting HLF might cause the upregulation of HLF in OC. Luciferase reporter assay identified miR-520e as an upstream regulator of HLF that directly targets HLF. Expression analysis further documented a negative correlation between miR-520e and HLF in OC, implying that miR-520e may be crucial for OC tumorigenesis. To the best of our knowledge, the potential role of miR-520e in the regulation of OC progression has not been reported. Dysregulated miR-520e has been reported to function as either an oncogene or a tumor suppressor, depending on the context [38, 39]. In the present study, we demonstrated that miR-520e inhibits OC cell self-renewal, proliferation, and metastasis by targeting HLF/YAP1. Furthermore, our clinical investigation showed that the combination of low miR-520e, high HLF, and nuclear accumulation of YAP1 predicted a worse prognosis than either marker alone, suggesting superior accuracy of the combined markers in evaluating the prognosis of OC patients. These findings not only provide a new mechanism of OC progression but also implicate a crucial role of the miR-520e/HLF/YAP1 axis in OC.

Carboplatin is a widely used chemotherapy drug for OC despite its significant toxic side effects [40, 41]. However, most patients develop resistance to carboplatin-based chemotherapy. Thus, there is an urgent need to elucidate the underlying mechanisms of carboplatin resistance and discover reliable biomarkers that can predict drug response in OC patients. Previous studies have shown that cells overexpressing HLF are resistant to chemotherapy. In the current study, we found that carboplatin resistance was mediated by HLF in OC cells. Moreover, our data demonstrated that carboplatin resistance in HLF-overexpressing OC cells could be abolished by the loss of YAP1, suggesting that the HLF/YAP1 axis is useful in determining carboplatin response. In addition, we found that HLF-depletion or the YAP1 inhibitor verteporfin could re-sensitize the carboplatin response of carboplatin-resistant OC cells, indicating that targeting HLF or YAP1 could be an optimal combinational therapeutic strategy to overcome carboplatin resistance in a subset of OC. Therefore, it is advisable to evaluate HLF or YAP1 expression in OC tumors to identify patients who might benefit from carboplatin therapy before deciding the course of treatment. This merits further investigation in biomarker-guided clinical trials.

In summary, our findings demonstrate that miR-520e/HLF/YAP1 signaling promotes OC progression and chemoresistance and that targeting this signaling pathway is a potential therapeutic strategy for OC.

## Materials and methods

### Human tissue samples

Thirty OC and 12 normal tissue samples were obtained from the First Affiliated Hospital of China Medical University. Detailed clinicopathological features of the patients are described in Supplementary Table S1. High-density TMA of human OC clinical samples (cat. no. HOvaC154Su01) were obtained from a cohort of 152 patients and constructed by Shanghai Outdo Biotech Co. (Shanghai, China). Detailed clinicopathological features of the patients are described in Supplementary Table S2. OS is defined as the time from the date of surgery to death. DFS is defined as the time from surgery to cancer relapse. If relapse was not diagnosed, patients were censored on the date of last follow-up or death. This study was approved by the Ethics Committee of the First Affiliated Hospital of China Medical University, and written informed consent was obtained from all included patients.

### Cell lines and viruses

Human OC cell lines (A2780, HO8910, and OVCAR3) were purchased from the Cell Bank of the Chinese Academy of Sciences (Shanghai, China). All human cell lines have been authenticated using STR profiling. Cells were cultured in Dulbecco’s modified Eagle medium (DMEM, Invitrogen) supplemented with 10% fetal bovine serum (FBS, Gibco) and 1% penicillin/streptomycin (HyClone); the incubation was carried out at 37 °C in a 5% CO_2_ atmosphere. HLF knockdown; YAP1 knockdown; control lentiviruses (designated as shHLF, shYAP1, and shCtrl); and lentiviruses expressing Flag-HLF, Flag-YAP1, or Flag (designated as HLF, YAP1, and Control) were obtained from Obio Technology Co. (Shanghai, China). The miR-520e sponge and mimic viruses were purchased from GenePharma (Shanghai, China). siRNA sequences are listed in Supplementary Table S7.

In order to establish carboplatin-resistant cell lines, A2780, HO8910, and OVCAR3 cells were incubated with carboplatin (Selleck, S1215) at a concentration just below their IC_50_; the concentration of carboplatin was gradually increased by 0.2 μM/L per week for 6–7 months. Three carboplatin-resistant cell lines, A2780CR, HO8910CR, and OVCAR3CR, were obtained. These carboplatin-resistant cell lines were maintained by continuous culture in the presence of carboplatin.

#### Spheroid formation assay

The spheroid formation assay was performed as described previously [42]. Briefly, cells from each group were counted and seeded in 96-well Ultra-Low Attachment Microplates (Corning, NY, USA) and cultured in DMEM/F12 (Invitrogen) supplemented with 1% FBS, 20 mg/mL bFGF (Invitrogen), and 20 ng/ml EGF (Peprotech). Spheres were photographed and counted after 7 days of culture.

#### In vitro limiting dilution assay

Serial numbers (64, 32, 26, 8, 4, and 2) of OC cells were seeded in 8 replicates in 96-well ultralow attachment culture plates. CSC proportions were analyzed using L-Calc software (Stem Cell Technologies, Inc.). The frequency of CSCs was assessed using ELDA software (https://bioinf.wehi.edu.au/software/elda/index.html) provided by the Walter and Eliza Hall Institute. In brief, limiting dilution analysis (LDA) accepts an input data table of three or four columns, separated by any combination of commas, spaces, or tabs. The data directly type into the webpage text field, or you can simply cut and paste the whole table from any spreadsheet application. Each row of data gives results for a particular cell dose. The columns are: 1. Dose: number of cells in each culture; 2. Tested: number of cultures tested; 3. Response: number of positive cultures; 4. Group (optional): a label for the population group to which cells belong. By default, ELDA computes a 95% confidence for the active cell frequency in each population group.

#### Cell proliferation assays

For the Cell Counting Kit-8 (CCK8) assay, OC and control cells were counted and seeded at 3 × 10^3^ cells in 96-well plates. CCK8 (MedChemExpress) (10 µl) was added to each well following the manufacturer’s instructions and incubated for 1 h. The absorbance at 450 nm was measured at the indicated time points using a microplate reader (Synergy H1; BioTek Instruments, Inc.).

For the colony formation assay, OC cells from each group were counted and seeded at 3 × 10^3^ cells in 12-well plates for 7 days. Cells were stained with crystal violet (Beyotime), and typical views were photographed.

For 5-ethynyl-2’-deoxyuridine (EdU) assay, OC cells of each group were counted and seeded at 3 × 10^3^ cells in 96-well plates for 2 days. Cells were stained with the EdU Kit (RiboBio) according to the manufacturer’s instructions, and typical views were photographed.

#### Cell migration and invasion assay

For the Transwell assay, OC cells from each group were counted and seeded at 2 × 10^5^ cells per well in a polycarbonate Transwell chamber in serum-free DMEM. The lower chamber of the 24-well plate contained DMEM supplemented with 20% FBS. After 24 h of incubation, the chambers were fixed and stained with crystal violet. Typical views were captured, and the corresponding cells were counted.

For the Matrigel invasion chamber assay, OC and control cells were counted and seeded at 2 × 10^5^ cells per well in a Boyden chamber coated with Matrigel (BD Biosciences) in serum-free DMEM. The lower chamber of the 24-well plate contained DMEM supplemented with 20% FBS. After 36 h of incubation, the cells were fixed and stained with crystal violet. Typical views were captured, and the corresponding cells were counted.

#### Experimental animal models

Female BALB/c nude mice (5–6 weeks of age, 18–20 g) and NOD-SCID mice (5–6 weeks of age, 22–25 g) were purchased from the Chinese Academy of Sciences Slack Company (Shanghai, China). All animals were dealt with according to the Animal Ethics Committee of China Medical University. Before tumor cell inoculation, mice were randomized into different groups. The investigator was blinded to the group allocation of the animals during the experiment. No statistical method was used to predetermine the sample size for the xenograft mice experiment, which was based on previous experimental observations. The sample size of each experiment is shown in the legend. No data were excluded from the analysis.

For in vivo limiting dilution assay, cells from each group were cultured under ultra-low attachment conditions for 7 days. The cell spheres were digested into single cells and counted. Indicated numbers of OC cells (1 × 10^3^, 1 × 10^4^, 1 × 10^5^, 1 × 10^6^) were mixed with Matrigel (1:1) and subcutaneously injected into NOD-SCID mice (*n* = 6 per group). The mice were sacrificed at 6 weeks, and the tumor incidence was calculated.

For in vivo tumor growth assay, 2 × 10^6^ cells from each group were subcutaneously injected into female BALB/c nude mice (*n* = 6 per group). The mice were sacrificed 8 weeks after injection. The weights of the xenografts were determined.

#### Mouse xenografts

A2780CR cells (5 × 10^6^ cells per mouse) were injected subcutaneously into the right posterior flank of 8-week-old female BALB/c nude mice (five mice per group). Tumor volumes were measured using a caliper twice a week using the following formula: volume = (width^2^ × length) × 0.5, where *L* is the longest tumor axis, and *W* is the shortest tumor axis. After tumor establishment, mice were intraperitoneally administered vehicle or carboplatin (Selleck, S1215, 60 mg/kg) every 2 days for up to 16 days (*n* = 5 for each group).

#### Patient-derived xenografts

For the patient-derived xenograft (PDX) model, primary tumor samples were obtained for xenograft establishment, as described previously [42]. When the PDX volume reached approximately 150 mm^3^, the female BALB/c nude mice were randomly assigned to treatment with vehicle, carboplatin (60 mg/kg, intraperitoneal injection once every other day), verteporfin (Selleck, S1786, 100 mg/kg, intraperitoneal injection once every other day), or a drug combination in which each compound was administered at the same dose and schedule as the signal agent (*n* = 5 per group) for 16 days. Tumor volumes were measured twice a week by employing a caliper, and the measurements were calculated using the following formula: volume = (width^2^ × length) × 0.5, where *L* is the longest tumor axis, and *W* is the shortest tumor axis. Mice were euthanized using CO_2_, and the tumors were sectioned or frozen for subsequent analysis. All procedures involving animals were performed in accordance with the ethical standards of the institutional and/or national research committee and the 1964 Helsinki Declaration and its later amendments or comparable ethical standards. All procedures and animal handling complied with animal welfare recommendations, and all animal study protocols were approved by the Ethical Committee of the First Affiliated Hospital of China Medical University.

### Western blotting assays

Protein extracts of cell lysate or human OC samples were subjected to sodium dodecyl sulfate-polyacrylamide gel electrophoresis (SDS-PAGE) and then transferred to the nitrocellulose membrane. The protein band was incubated with primary antibodies and IRDye 800CW-conjugated second antibody on LI-COR imaging system (LI-COR Biosciences). The antibodies used are listed in Supplementary Table S6.

### Statistical analysis

Statistical analyses were performed using SPSS software (version 22.0; SPSS, Inc.). The data are expressed as the mean ± SD. Student’s *t*-tests were used to compare two variables. Multivariate analysis was performed using the Cox multivariate proportional hazard regression model in a stepwise manner (forward: likelihood ratio). The relationship between the two variables was analyzed using the Pearson correlation method. A two-sided *p*-value less than 0.05 was considered statistically significant.

The remainder of the description of the materials and methods can be found in the Supplementary materials.

## Supplementary information

Supplemental Figures and Table

author-contribution-form

Original Data File

aj-checklist

## Data Availability

The data in the current study are available from the corresponding authors upon reasonable request.
