# Supplementary material for: HLF promotes ovarian cancer progression and chemoresistance via regulating Hippo signaling pathway
Source: Cell Death Dis. 2023 Sep 14;14(9):606. doi: 10.1038/s41419-023-06076-5 (PMC10502110; doi:10.1038/s41419-023-06076-5)
Supplement: Supplementary file 1 — Supplemental Figures and Table [file 41419_2023_6076_MOESM1_ESM.docx]

**HLF promotes ovarian cancer progression and chemoresistance** **via regulating Hippo signaling pathway**

Tao Han^1,#^, Tingsong Chen^2,#^, Lujun Chen^1,3#^, Kexin Li^1^, Daimin Xiang^4,5^, Lei Dou^6,*^, Hengyu Li^7,*^, Yubei Gu^8,*^

^1^Department of Oncology, the First Affiliated Hospital of China Medical University, Shenyang 110001, Liaoning, China

^2^Department of Cancer Intervention, Seventh People's Hospital of Shanghai University of TCM. Shanghai, 200001, China

^3^Postgraduate College, China Medical University, Shenyang 110001, China

^4^Clinical Cancer Institute, Center for Translational Medicine, Naval Military Medical University, Shanghai, 200433, China

^5^Department of hepatobiliary surgery, East Hospital, School of Medicine, Tongji University, Shanghai, 200120, China

^6^Department of Gynecology, the First Affiliated Hospital of China Medical University, Shenyang 110001, Liaoning, China

^7^Department of Breast and Thyroid Surgery, Changhai Hospital, Naval Military Medical University, Shanghai 200433, China

^8^Department of Gastroenterology, Ruijin Hospital Affiliated to Shanghai Jiao Tong University School of Medicine, Shanghai, China

^#^These authors contributed equally to this work.

***Correspondence author address:**

Dr. Yubei Gu ([gyb11809@rjh.com.cn](mailto:200025gyb11809@rjh.com.cn)), Dr. Hengyu Li ([lhy@smmu.edu.cn](mailto:lhy@smmu.edu.cn)) and Dr. Lei Dou (doulei840416@163.com). Department of Gastroenterology, Ruijin Hospital Affiliated to Shanghai Jiao Tong University School of Medicine, Shanghai, China.

**Running Head:** HLF facilitates OC development

**Materials and Methods**

**Flow Cytometric Analysis of ALDH Activity**

ALDH activity was assessed using the ALDEFLUOR kit (Stem Cell Technologies, Durham, NC, USA). Briefly, cells (4×10^4^/well) were incubated with ALDH substrate BAAA for 30 min at 37 °C following the manufacturer’s instructions. Cells treated with diethyl-aminobenzaldehyde (DEAB), a specific ALDH inhibitor, were used as a control to establish the baseline fluorescence and define the cut-off for ALDEFLUOR-positive cells.

**Chromatin immunoprecipitation (ChIP) assay**

ChIP assay was carried out in HO8910 and A2780 cells using an EpiTect ChIP qPCR Kit (QIAGEN) as described previously (1). In brief, 1×10^7 cells fixed with formaldehyde were collected and added with 500ul lysis buffer. Then lysate was sonicated for 25 cycles of 6-s power-on and 30-s interval with intensity of 200 W. Next, the supernatants were mixed with Protein A/G magnetic beads. Then chromatin was immunoprecipitated with IgG or anti-Flag antibodies overnight. The next day, the mixture was washed and incubated with elution buffer at 62 °C for 2 h and then at 95 °C for 10 min. Then bound DNA was purified and complied to qPCR.

**Dual luciferase reporter gene assay**

DNA fragments of HLF-3'UTR containing the wildtype miR-520e motifs or mutant motifs (“AGCACUUU” to “UCUCAGAU”) were inserted downstream of firefly luciferase using pMIR-REPORT vector (Obio Technology, Shanghai). The DNA sequences containing the promoter region of YAP1 (−1900/+50) were cloned into pGL6-luc plasmid (designated as YAP1-WT). The potential HLF binding sites within the YAP1 promoter were further deleted in pGL6-YAP1-WT-luc respectively (designated as (−1300/+50) YAP1, (−700/+50) YAP1, (−200/+50) YAP1, (−1900/+50) YAP1). The HLF binding sites in the YAP1 promoter were mutated using the QuikChange II Site-Directed Mutagenesis Kit (Stratagene). The cells were transfected with one of the above plasmids and pRL-TK-Renilla-luc plasmids. For TOP/FOP flash reporter assay, the TOPflash reporter plasmids, containing wildtype TCF receptor, and FOPflash reporter plasmids, containing mutant TCF receptor, were obtained from Promega. Cells were co-transfected with TOPflash or FOPflash and pRL-TK-Renilla-luc plasmid. The luciferase reporter gene assay was performed as previously described (3). Briefly, firefly luciferase activity of each group in triplicate was analyzed by Dual-Luciferase Reporter Assay Kit (Promega) using a Synergy 2 Multidetection Microplate Reader (BioTek Instruments). Firefly luciferase activity were normalized to Renilla luciferase activity.

**Quantitative real-time polymerase chain reaction (qPCR)**

Total RNA was isolated from cells or tissues using TRIZOL (Invitrogen) according to the manufacturer’s instructions. The purity of RNA was measured with a UV spectrophotometer (NanoDrop ND-1000) and RNA integrity was validated with agarose gel electrophoresis. The extracted RNA was then reverse-transcribed to cDNA with the M-MLV RTase cDNA Synthesis Kit (Promega). Real-time PCR analysis was performed using a SYBR Green PCR Kit (Roche) and LightCycler 480 System (Roche). PCR conditions included 1 cycle at 95 °C for 5 minutes, followed by up to 40 cycles of 95 °C for 15 seconds (denaturation), 60 °C for 30 seconds (annealing) and 72 °C for 30 seconds (extension). The sequences for primers are listed in Supplementary Table S7.

**Immunohistochemical (IHC) staining**

IHC staining were described previously (2). Briefly, tissue samples or xenografts were formalin-fixed paraffin-embedded (FFPE), and sectioned for following IHC examination. Sections were de-paraffined in dimethyl benzene and ladder concentration of ethanol (100%, 95%, 85%, 75%), heated for antigen retrieval, blocked by 3% H_2_O_2_ and 1% BSA, incubated with primary antibody and secondary antibody, and colored with diaminobenzidine (DAB). The stained sections were scanned using ScanScope XT scanner (Aperio Technologies, Inc.). The quality of immunostaining was independently evaluated by two pathologists blindly. The high-resolution digital images scored by a pathologist using ‘positive pixel count v9’ algorithms provided by Image Scope program (Aperio Technologies, Inc.). Briefly, the program automatically counted pixels and measured the intensity of positive (brown staining) pixels. The staining score for each sample was defined as average positivity (sum intensity of positive pixels per total positive pixels) times positivity (number of positive pixels per total pixels). The median HLF, YAP1 and miR-520e score was set as cut-off value to divide the cohort into high and low subgroup.

**Immunofluorescence staining**

Cells were fixed with 4% formalin for 15 min at room temperature. After incubated with 0.1% Triton X-100 for 5 min, cells were blocked with 10% goat serum for 1 hour. Cells were incubated with primary antibody (HLF and YAP1) at 4 ℃ overnight and secondary antibody (Goat anti-Mouse IgG Alexa Fluor 488 and Goat anti-Rabbit IgG Alexa Fluor 594 from Life Technologies) for 30 min at room temperature. Cells were then stained with 4′,6-diamidino-2-phenylindole (DAPI) (Sigma) and observed under fluorescent/confocal microscope (Olympus).

**Apoptosis assay**

The apoptosis of hepatoma cells was measured by flow cytometry using a FITC Annexin V Apoptosis Detection Kit (BD Biosciences 51-66211E). Briefly, 1×106 cells were harvested and washed twice with cold cell staining buffer, resuspended in 100 μL Annexin V binding buffer, then incubated with 5 μL of APC Annexin V and 5 μL of 7-AAD viability staining solution for 15 minutes at room temperature in the dark. The cell suspension was then incubated with 400 μL of Annexin V binding buffer followed by flow cytometry analysis.

**Reference:**

1. Xiang D, Cheng Z, Liu H, Wang X, Han T, Sun W, et al. Shp2 promotes liver cancer stem cell expansion by augmenting β-catenin signaling and predicts chemotherapeutic response of patients. Hepatology 2017 05;65(5)

2. Xiang DM, Sun W, Zhou T, Zhang C, Cheng Z, Li SC, et al. Oncofetal HLF transactivates c-Jun to promote hepatocellular carcinoma development and sorafenib resistance. Gut 2019 10;68(10)

3. Zhou T, Li S, Xiang D, Liu J, Sun W, Cui X, et al. m6A RNA methylation-mediated HNF3γ reduction renders hepatocellular carcinoma dedifferentiation and sorafenib resistance. Signal Transduct Target Ther 2020 12 26;5(1)


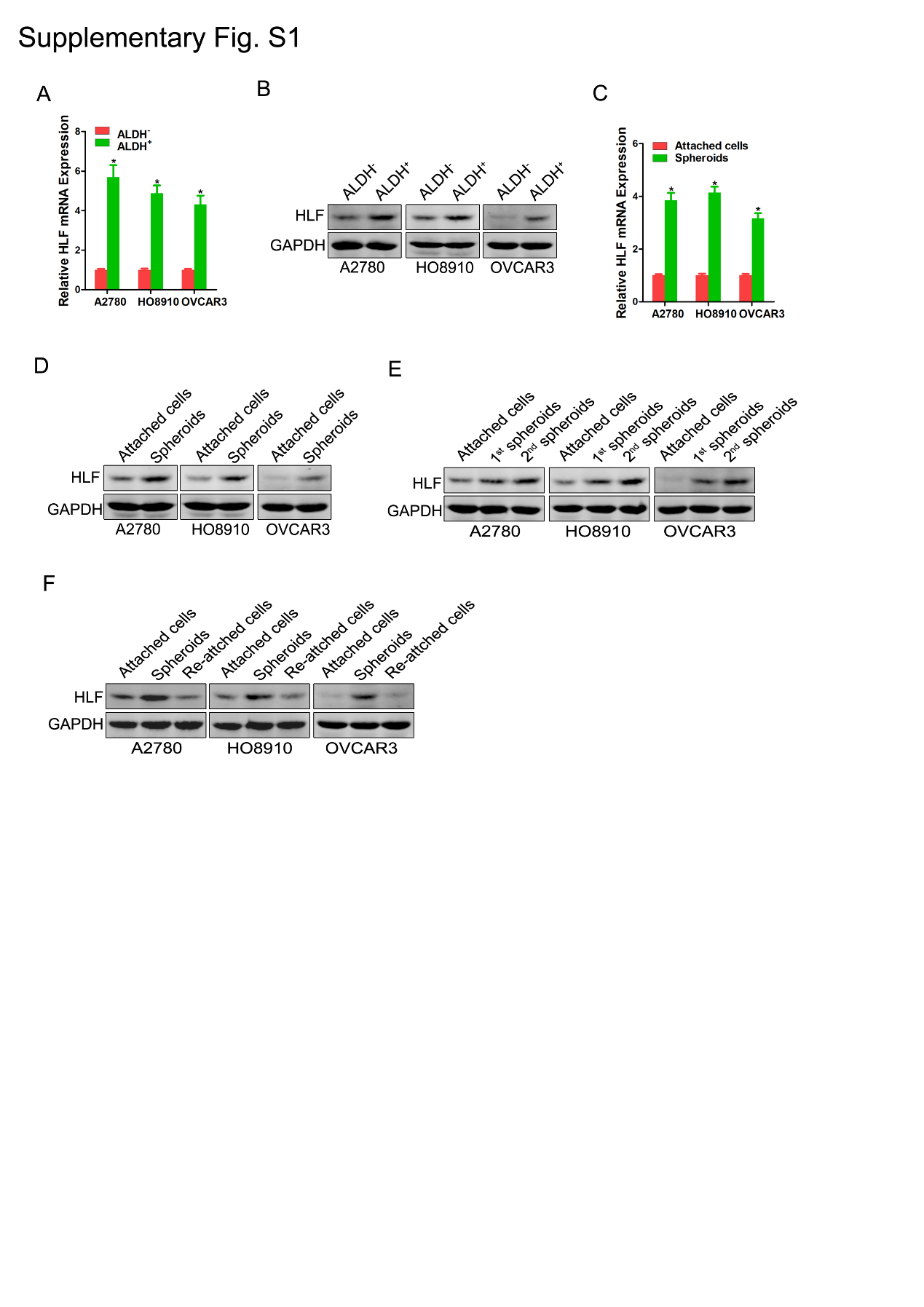


**Supplementary Fig. S1**

**A.** Real-time PCR analysis of HLF expression in flow sorted ALDH^+^ and ALDH^-^ OC cells.

**B.** Western blot analysis of HLF expression in flow sorted ALDH^+^ and ALDH^-^ OC cells.

**C.** Real-time PCR analysis of HLF expression in OC adherent cells and spheres.

**D.** Western blot analysis of HLF expression in OC adherent cells and spheres.

**E.** Western blot analysis of HLF expression in serial passages of OC cell spheroids.

**F.** Western blot analysis of HLF expression HLF expression in OC adherent cells, spheres and reattached cells.


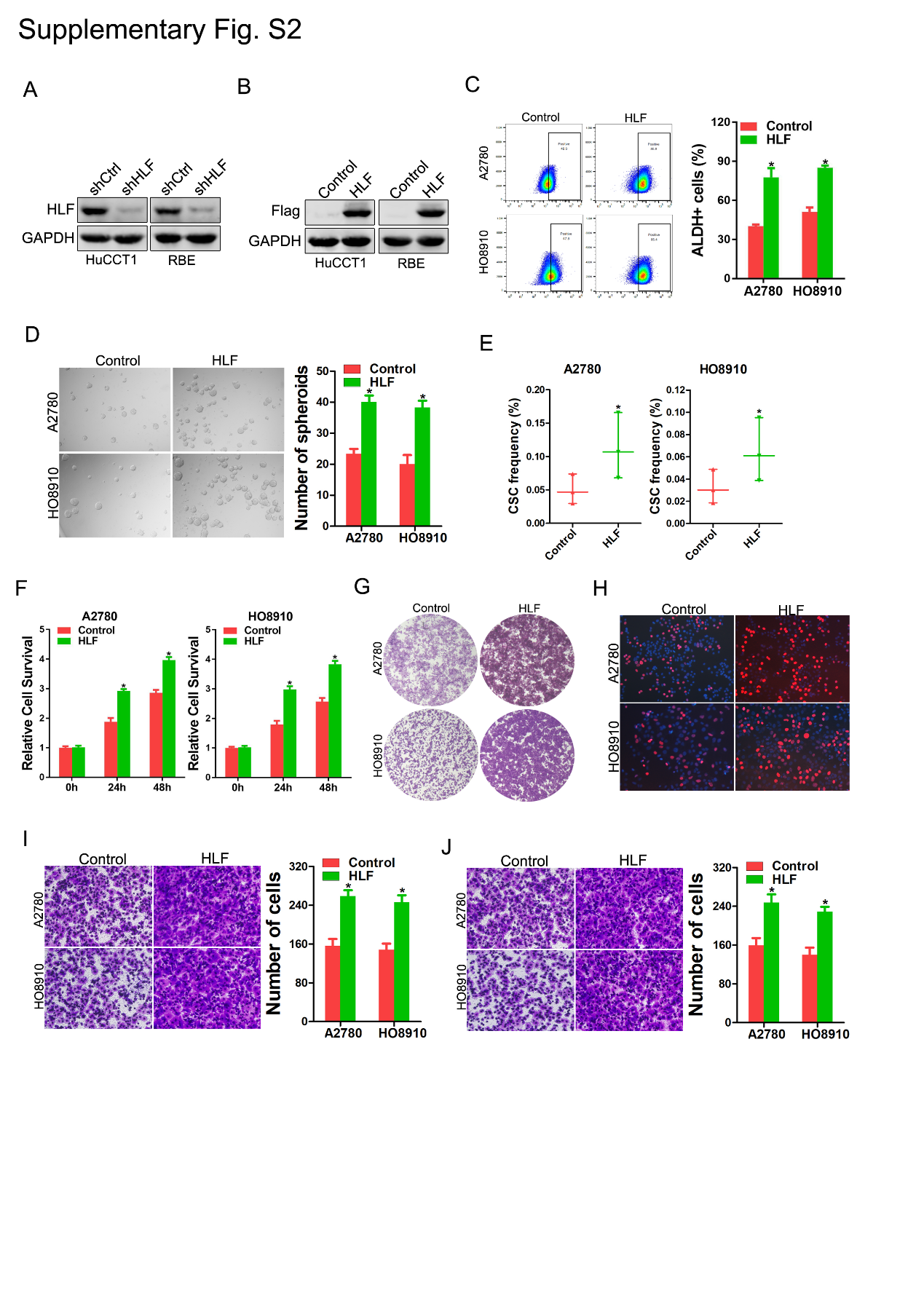


**Supplementary Fig. S2**

**A.** Western blot analysis of HLF expression in HLF knockdown and control OC cells.

**B.** Western blot analysis of HLF expression in HLF overexpression and control OC cells.

**C.** Flow cytometry analysis of ALDH^+^ populations in HLF overexpression and control OC cells.

**D.** Representative images of spheroids generated from HLF overexpression and control OC cells. The number of spheroids was counted and compared.

**E.** The frequency of CSCs in HLF overexpression and control OC cells was compared by *in vitro* limiting dilution assay.

**F.** Proliferation of HLF overexpression and control OC cells was evaluated by Cell Counting Kit 8 Assay.

**G.** HLF overexpression or control OC cells were subjected to the colony formation assay. The formed colonies were fixed and stained with crystal violet, and representative images were shown.

**H.** Representative images of EdU staining of proliferating HLF overexpression or control OC cells. EdU^+^ cells were stained with red immunofluorescence. The nuclei were counterstained with DAPI. Scale bar=50 μm.

**I.** Migration assay was performed utilizing polycarbonate membrane inserts in a 24-well plate.

**J.** The invasive properties of HLF overexpression or control OC cells were analyzed using Matrigel-coated Boyden chamber.


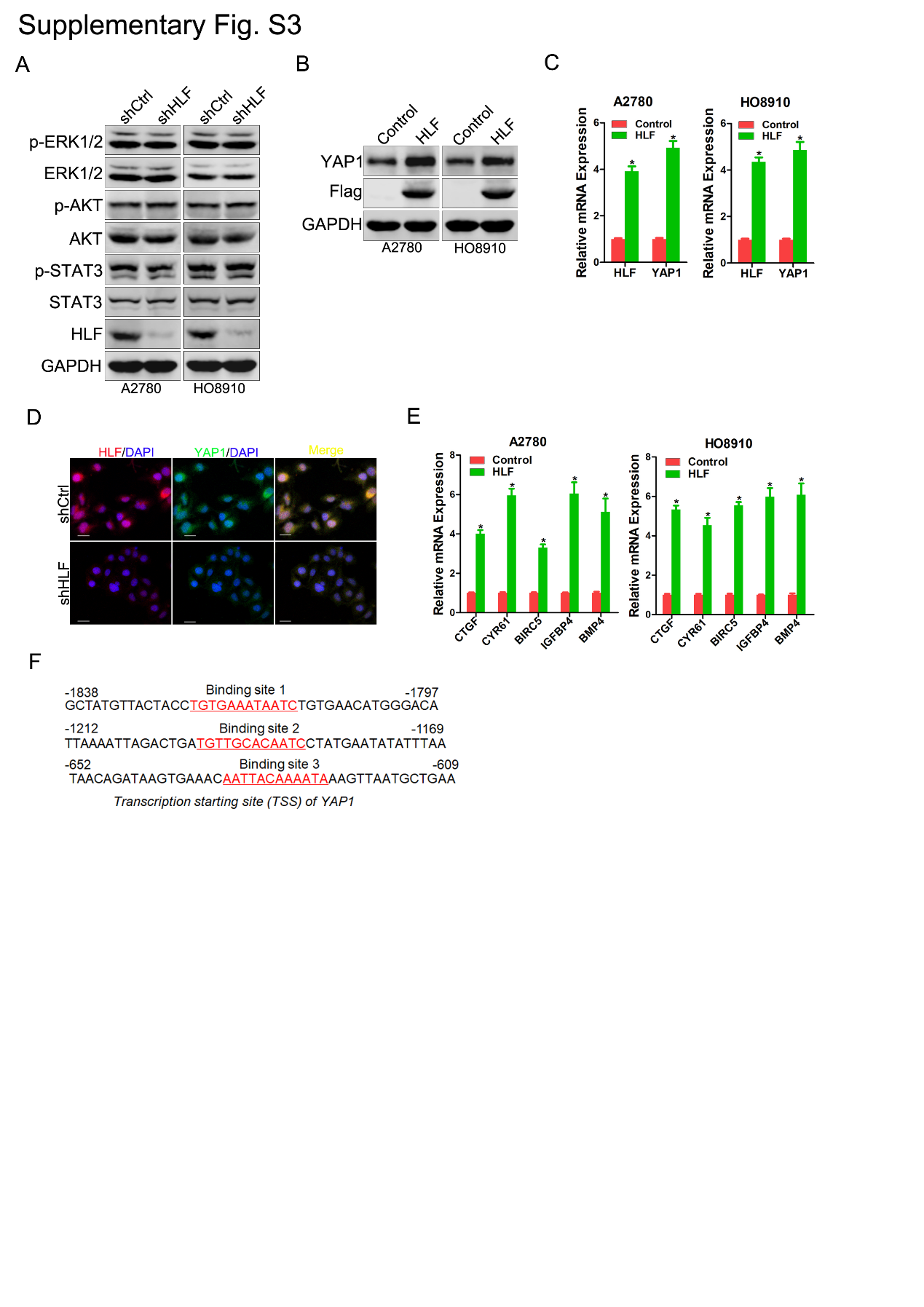


**Supplementary Fig. S3**

**A.** Western blot analysis of indicated protein expression in HLF knockdown and control OC cells.

**B.** Western blot analysis of the protein expression of HLF and YAP1 in HLF overexpression or control OC cells.

**C.** Real-time PCR analysis of the mRNA expression of HLF and YAP1 in HLF overexpression or control OC cells.

**D.** Representative images of dual immunofluorescence staining of HLF and YAP1 in HO8910 shHLF or control cells. The nuclei were counterstained with DAPI. Scale bar=20 μm.

**E.** Real-time PCR analysis of the mRNA expression of YAP1 targeted genes in HLF overexpression control OC cells.

**F.** Potential binding sites of HLF in YAP1 promoter.


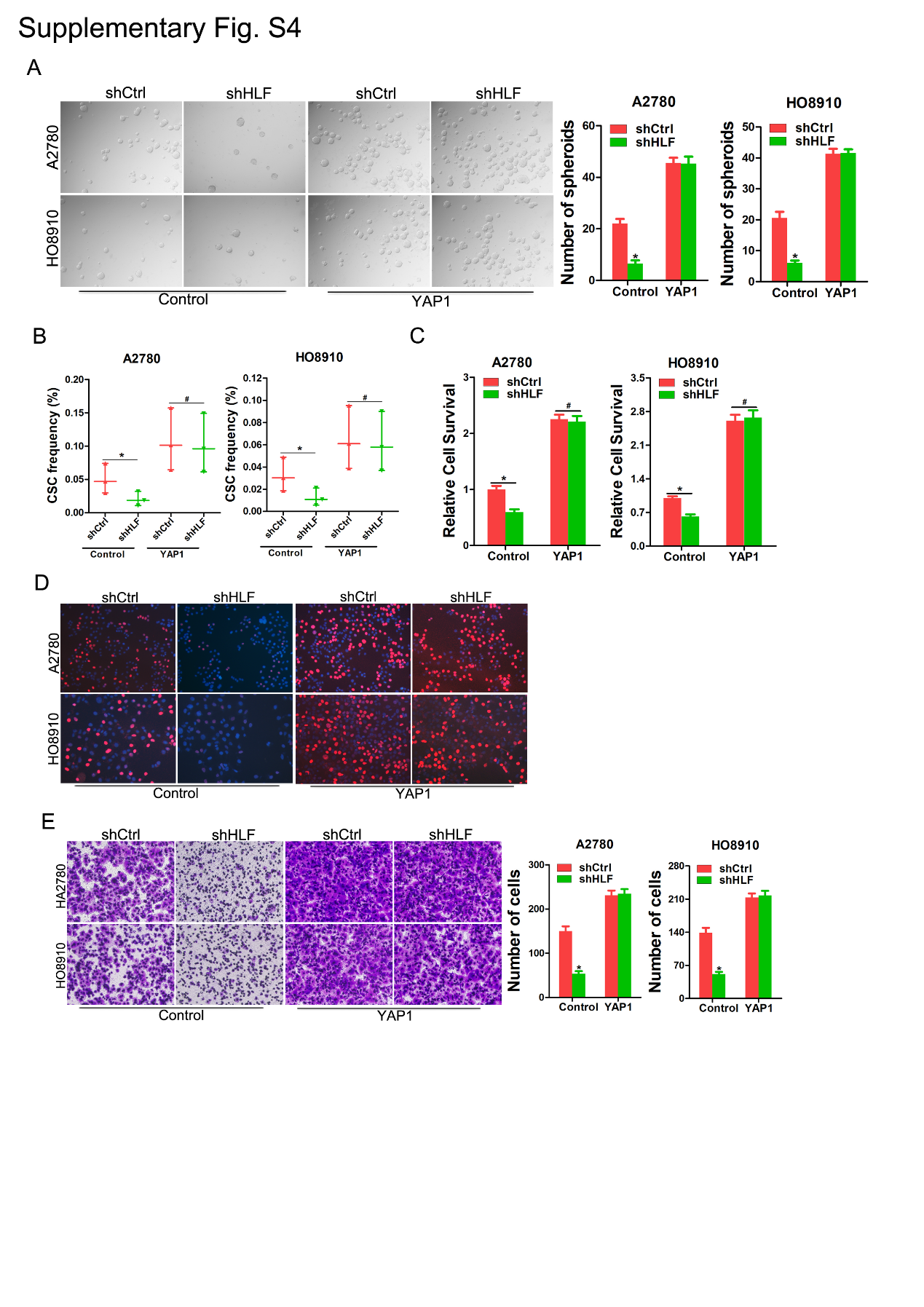


**Supplementary Fig. S4**

**A.** A2780/HO8910 shHLF and control cells infected with YAP1 overexpression or control virus were subjected to spheroids formation.

**B.** A2780/HO8910 shHLF and control cells infected with YAP1 overexpression or control virus were subjected to *in vitro* limiting dilution assay.

**C.** A2780/HO8910 shHLF and control cells infected with YAP1 overexpression or control virus were subjected to CCK-8 analysis.

**D.** A2780/HO8910 shHLF and control cells infected with YAP1 overexpression or control virus were subjected to EdU staining.

**E.** A2780/HO8910 shHLF and control cells infected with YAP1 overexpression or control virus were subjected to Matrigel invasion chamber assay.


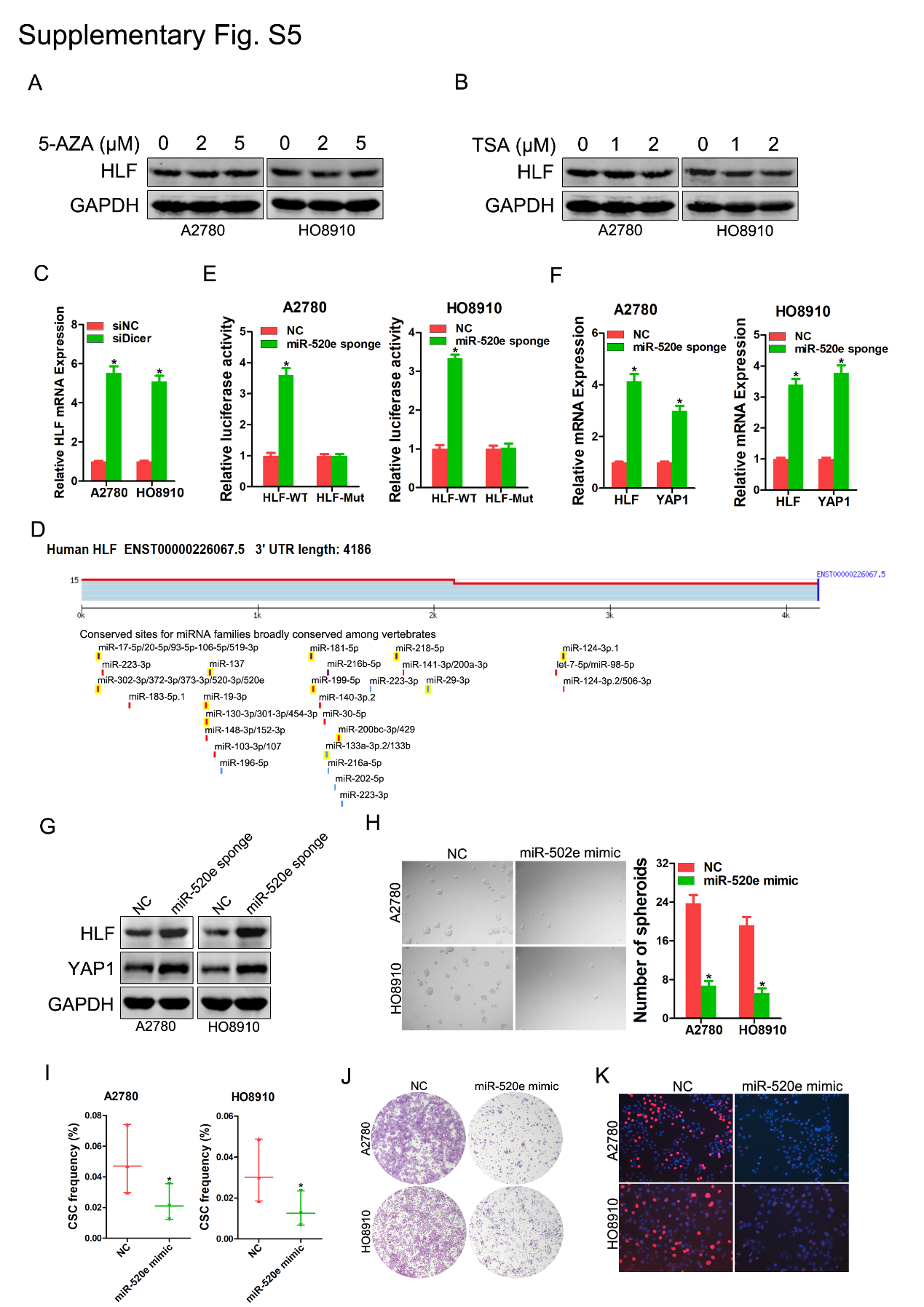

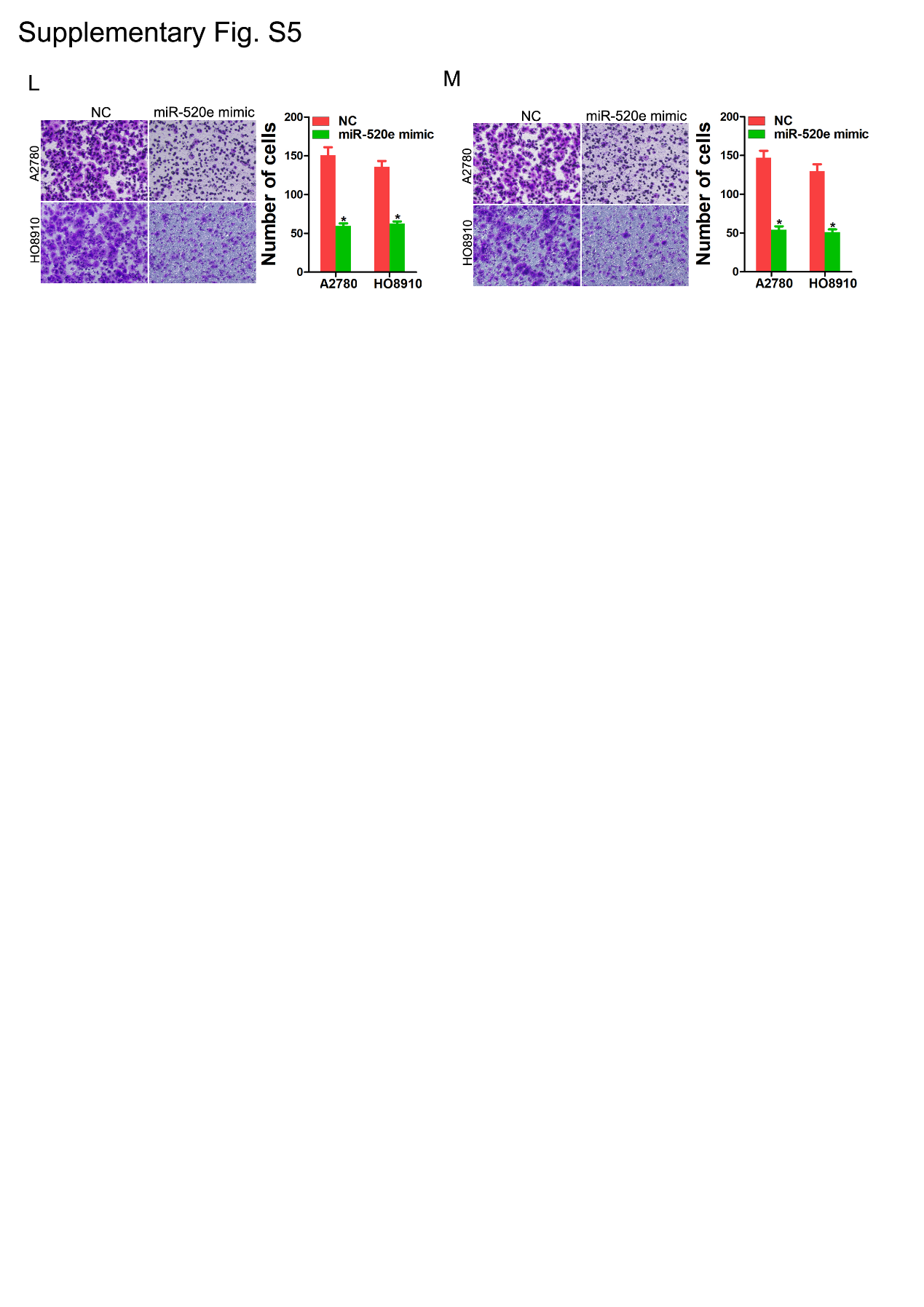


**Supplementary Fig. S5**

**A.** OC cells were treated with 5-Aza-CdR for 24 hours followed by western blot analysis.

**B.** OC cells were treated TSA for 24 hours followed by western blot analysis.

**C.** OC cells were transfected with siDicer or siNC for 24 hours followed by real-time PCR analysis.

**D.** The scheme of the structure of the HLF gene 3’UTR and the relative binding of miRNAs. (https://www.targetscan.org/vert_80/)

**E.** Luciferase reporter assays performed in miR-520e sponge and control OC cells transfected with wild-type or mutant HLF 3’UTR constructs.

**F.** Real-time PCR analysis mRNA expression of HLF and YAP1 in miR-520e sponge and control OC cells.

**G.** Western blot analysis protein expression of HLF and YAP1 in miR-520e sponge and control OC cells.

**H.** Representative images of spheroids generated from miR-520e sponge and control OC cells. The number of spheroids was counted and compared.

**I.** The frequency of CSCs in miR-520e sponge and control OC cells was compared by *in vitro* limiting dilution assay.

**J.** miR-520e sponge or control OC cells were subjected to the colony formation assay. The formed colonies were fixed and stained with crystal violet, and representative images were shown.

**K.** Representative images of EdU staining of proliferating miR-520e sponge or control OC cells. EdU^+^ cells were stained with red immunofluorescence. The nuclei were counterstained with DAPI. Scale bar=50 μm.

**L.** Migration assay was performed utilizing polycarbonate membrane inserts in a 24-well plate.

**M.** The invasive properties of miR-520e sponge or control OC cells were analyzed using Matrigel-coated Boyden chamber.


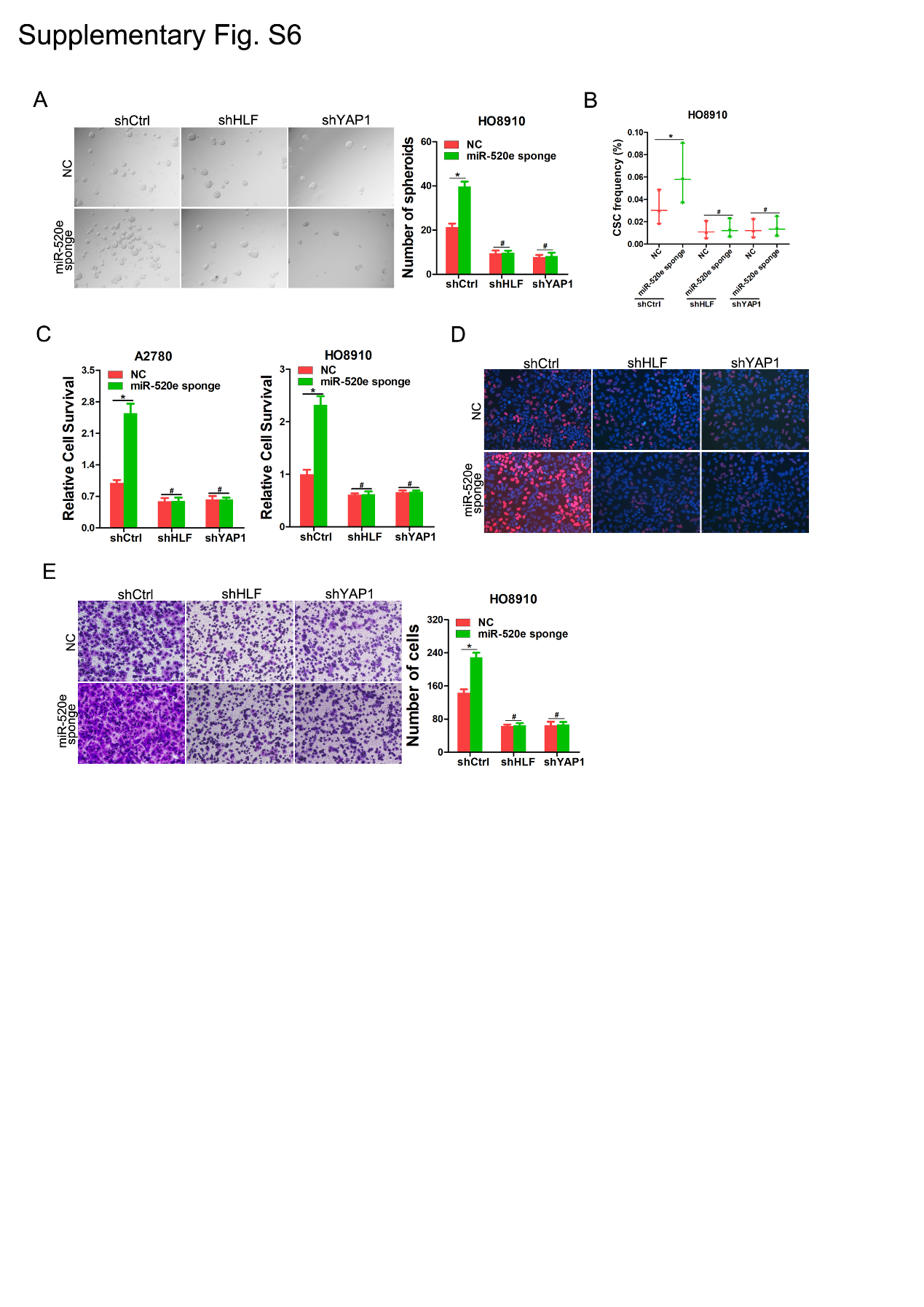


**Supplementary Fig. S6**

**A.** HO8910 miR-520e sponge and control cells infected with HLF or YAP1 knockdown virus were subjected to spheroids formation.

**B.** HO8910 miR-520e sponge and control cells infected with HLF or YAP1 knockdown virus were subjected to *in vitro* limiting dilution assay.

**C.** A2780/HO8910 miR-520e sponge and control cells infected with HLF or YAP1 knockdown virus were subjected to CCK-*8* assay.

**D.** HO8910 miR-520e sponge and control cells infected with HLF or YAP1 knockdown virus were subjected to EdU staining.

**E.** HO8910 miR-520e sponge and control cells infected with HLF or YAP1 knockdown virus were subjected to Matrigel invasion chamber assay.


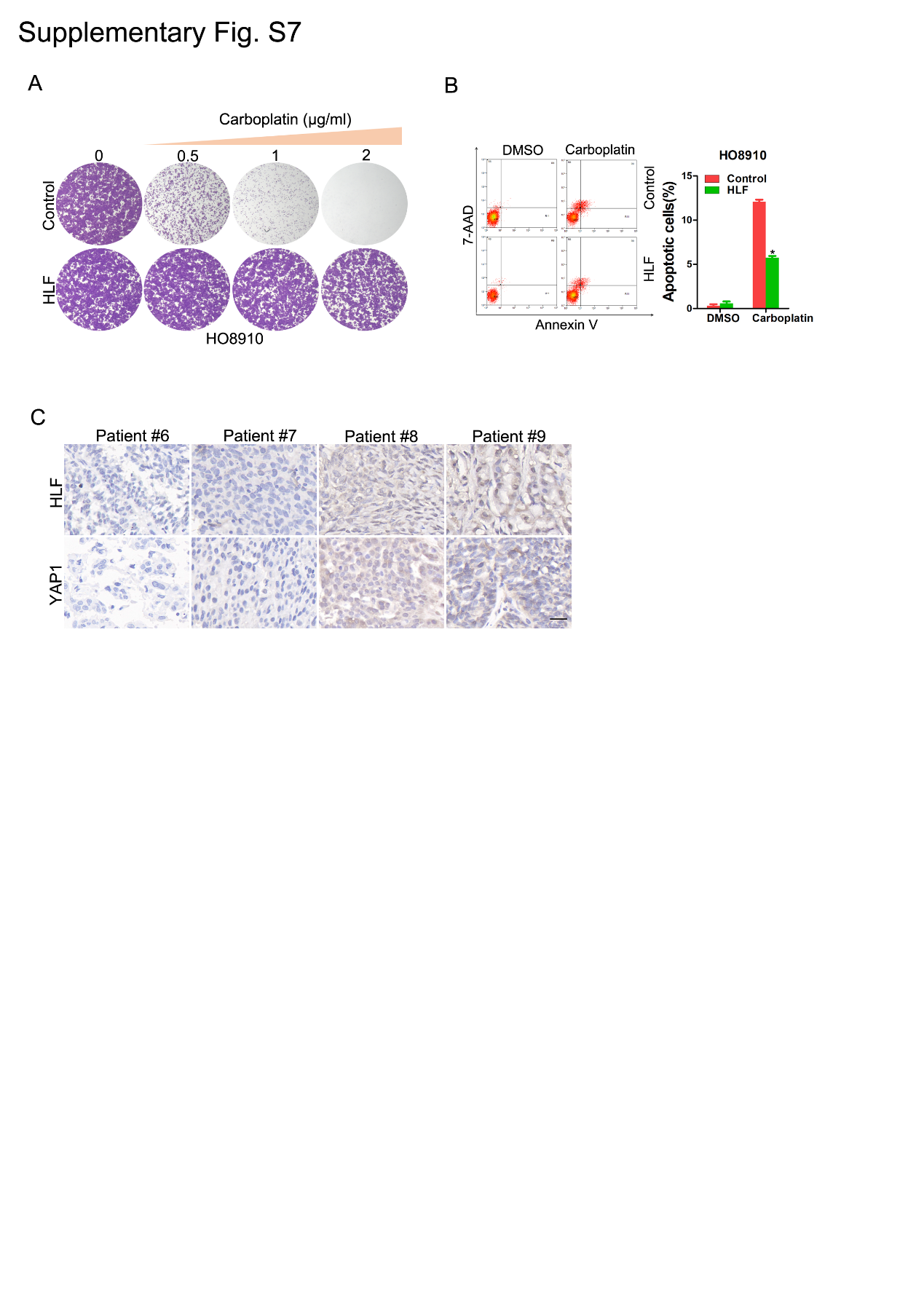


**Supplementary Fig. S7**

**A.** HO8910 HLF and control cells were treated with carboplatin for 10 days and their colony growth was examined.

**B.** HO8910 HLF and control cells were treated with carboplatin (4 μg/ml) for 48 hours and their apoptosis was examined by flow cytometry.

**C.** IHC staining of HLF and YAP1 in the primary OCs. Scale bar = 25μm.


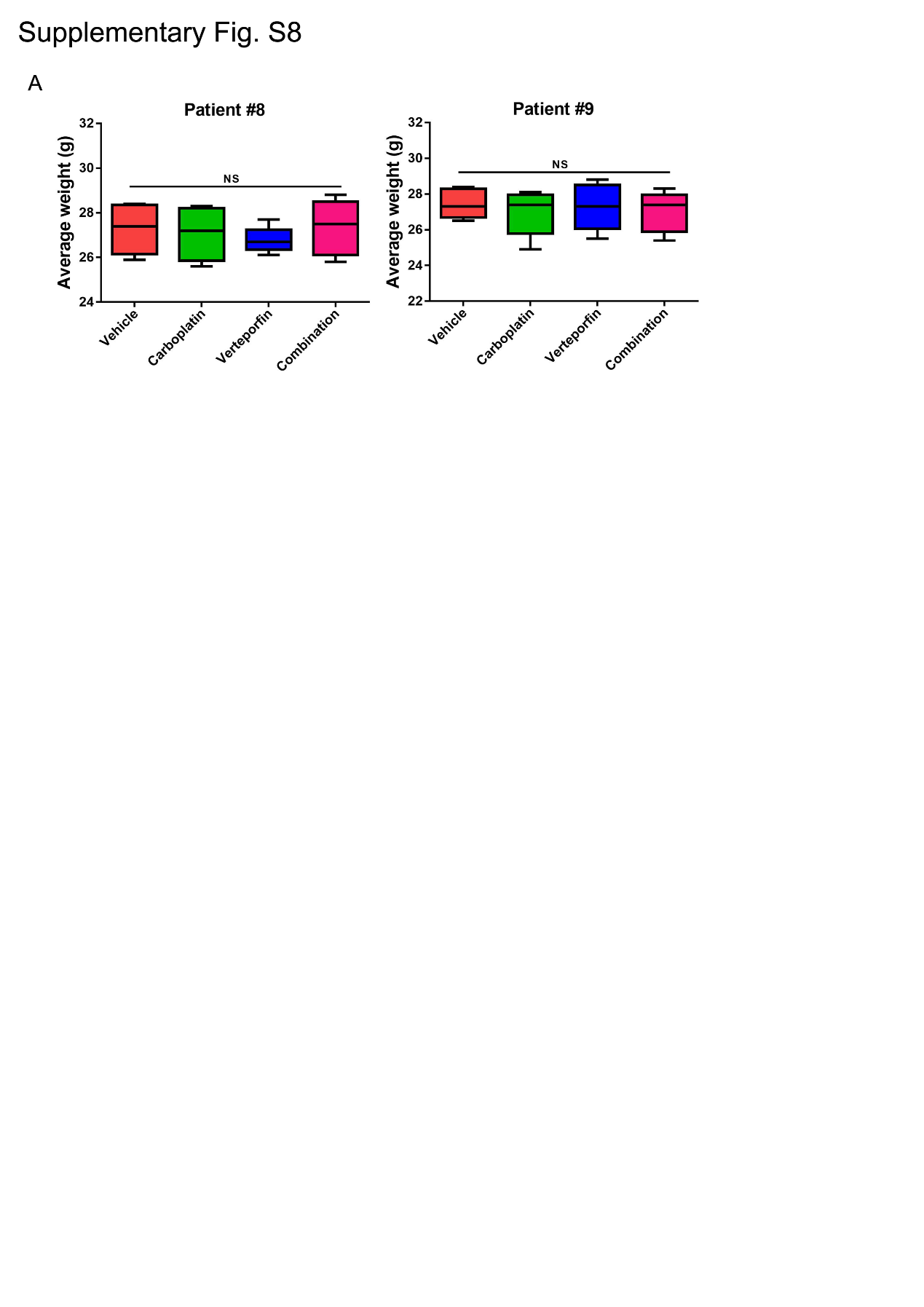


**Supplementary Fig. S8**

**A.** Average weight of mice (n = 5 per group) xenografted with PDXs after indicated treatments. The horizontal lines in the box plots represent the medians, the boxes represent the interquartile range, and the whiskers represent the minimum and maximum values.

**Supplementary Tables**

**Supplemental Table S1. Clinicopathologic features of 30 OC Specimens**

| Characteristics |  | n=30 |
| --- | --- | --- |
| Age(year) | ≤50 | 8 |
|  | >50 | 22 |
| Histological grade | I-II | 9 |
|  | III | 21 |
| T stage | 1-2 | 11 |
|  | 3 | 19 |
| N stage | 0 | 20 |
|  | 1 | 10 |
| M stage | 0 | 17 |
|  | 1 | 13 |
| TNM | I-II | 11 |
|  | III-IV | 19 |

TNM, Tumor-Nodes-Metastasis

**Supplementary Table S2. Clinicopathologic Features of** **152 OC Specimens in Cohort 1**

| Characteristics |  | |  | HLF low  (n=76) | HLF high  (n=76) | p value |
| --- | --- | --- | --- | --- | --- | --- |
| Age(year) | ≤50 | |  | 34 | 38 | p>0.05 |
|  | >50 |  | | 42 | 38 |  |
| Histological grade | I-II | | | 27 | 27 | p>0.05 |
|  | III | | | 49 | 49 |  |
| T stage | 1-2 | | | 26 | 18 | p>0.05 |
|  | 3 | | | 50 | 58 |  |
| N stage | 0 | |  | 65 | 47 | p<0.05 |
|  | 1 | |  | 11 | 29 |  |
| M stage | 0 | |  | 67 | 53 | p<0.05 |
|  | 1 | |  | 9 | 23 |  |
| TNM | I-II | |  | 26 | 18 | p>0.05 |
|  | III-IV | |  | 50 | 58 |  |

TNM, Tumor-Nodes-Metastasis

**Supplementary Table S3. Multivariate Cox regression analysis (Overall survival)**

| Characteristics | RC *P* HR 95% CI | | | |
| --- | --- | --- | --- | --- |
| TNM stage | 2.487 | ＜0.001 | 12.020 | 4.390-32.905 |
| HLF | 0.687 | 0.003 | 1.988 | 1.272-3.108 |

**Supplementary Table S4. Multivariate Cox regression analysis (Disease-free survival)**

| Characteristics | RC *P* HR 95% CI | | | |
| --- | --- | --- | --- | --- |
| TNM stage | 1.748 | ＜0.001 | 5.741 | 3.401-9.692 |
| HLF | 0.422 | 0.020 | 1.525 | 1.068-2.180 |

**Supplemental Table S5.** **Ninety-one conserved binding miRNAs upon HLF 3’UTR**

| hsa-miR-302a-3p | hsa-miR-302d-3p | hsa-miR-302c-3p.1 | hsa-miR-302b-3p | hsa-miR-302e |
| --- | --- | --- | --- | --- |
| hsa-miR-373-3p | hsa-miR-520e | hsa-miR-520d-3p | hsa-miR-520c-3p | hsa-miR-372-3p |
| hsa-miR-520b | hsa-miR-520a-3p | hsa-miR-106b-5p | hsa-miR-519d-3p | hsa-miR-20a-5p |
| hsa-miR-106a-5p | hsa-miR-93-5p | hsa-miR-17-5p | hsa-miR-20b-5p | hsa-miR-526b-3p |
| hsa-miR-223-3p | hsa-miR-183-5p.1 | hsa-miR-411-5p.2 | hsa-miR-19a-3p | hsa-miR-19b-3p |
| hsa-miR-454-3p | hsa-miR-130a-3p | hsa-miR-301a-3p | hsa-miR-4295 | hsa-miR-301b-3p |
| hsa-miR-130b-3p | hsa-miR-3666 | hsa-miR-148b-3p | hsa-miR-148a-3p | hsa-miR-152-3p |
| hsa-miR-137 | hsa-miR-1251-5p | hsa-miR-107 | hsa-miR-103a-3p | hsa-miR-196a-5p |
| hsa-miR-196b-5p | hsa-miR-181c-5p | hsa-miR-181d-5p | hsa-miR-181b-5p | hsa-miR-181a-5p |
| hsa-miR-4262 | hsa-miR-199a-5p | hsa-miR-199b-5p | hsa-miR-758-3p | hsa-miR-140-3p.2 |
| hsa-miR-30e-5p | hsa-miR-30a-5p | hsa-miR-30d-5p | hsa-miR-30c-5p | hsa-miR-30b-5p |
| hsa-miR-133b | hsa-miR-133a-3p.2 | hsa-miR-216b-5p | hsa-miR-216a-5p | hsa-miR-202-5p |
| hsa-miR-429 | hsa-miR-200c-3p | hsa-miR-200b-3p | hsa-miR-223-3p | hsa-miR-374a-5p |
| hsa-miR-369-3p | hsa-miR-374b-5p | hsa-miR-410-3p | hsa-miR-223-3p | hsa-miR-218-5p |
| hsa-miR-141-3p | hsa-miR-200a-3p | hsa-miR-29a-3p | hsa-miR-29c-3p | hsa-miR-29b-3p |
| hsa-miR-653-5p | hsa-miR-873-5p.2 | hsa-miR-4500 | hsa-let-7i-5p | hsa-let-7g-5p |
| hsa-let-7b-5p | hsa-let-7a-5p | hsa-miR-4458 | hsa-let-7c-5p | hsa-miR-98-5p |
| hsa-let-7e-5p | hsa-let-7f-5p | hsa-let-7d-5p | hsa-miR-124-3p.1 | hsa-miR-124-3p.2 |
| hsa-miR-506-3p |  |  |  |  |

**Supplementary Table S6. Antibody List.**

| **Antigens** | **Manufacturer** | **Application** |
| --- | --- | --- |
| HLF | Abcam (ab91630), USA | 1:1000 for WB |
| HLF | The International Cooperation Laboratory on Signal Transduction, EHBH, SMMU, China | 1:100 for IHC or 1:50 for IF |
| YAP1 | Proteintech Group (13584-1-AP), China | 1:1000 for WB or 1:100 for IHC or 1:50 for IF |
| PARP | Proteintech Group (13371-1-AP), China | 1:1000 for WB |
| Flag | Abcam, Cambridge (ab205606), MA | 1:1000 for WB or 1:50 for IP |
| p-STAT3 | Abcam, Cambridge (ab267373), MA | 1:1000 for WB |
| STAT3 | Proteintech Group (10253-2-AP), China | 1:1000 for WB |
| p-ERK1/2 | Abcam, Cambridge (ab176640), MA | 1:1000 for WB |
| ERK1/2 | Proteintech Group (11257-1-AP), China | 1:1000 for WB |
| p-AKT | Abmart (T40067), China | 1:1000 for WB |
| AKT | Proteintech Group (60203-2-Ig), China | 1:1000 for WB |
| GAPDH | Proteintech Group (60004-1-Ig), China | 1:5000 for WB |

**Supplementary Table S7. Primer List.**

| **Gene** | **Forward primer (5’-3’)** | **Reverse primer (5’-3’)** | |
| --- | --- | --- | --- |
| HLF(Human) | Forward (5*′*- 3*′*) | ACCAAGTCCCATTGATCCTG | |
|  | Reverse (5*′*- 3*′*) | GCCCAGTACTTGTCATCCTTC | |
| β-actin(Human) | Forward (5*′*- 3*′*) | TGGCACCCAGCACAATGAA | |
|  | Reverse (5*′*- 3*′*) | CTAAGTCATAGTCCGCCTAGAAGCA | |
| YAP1(Human) | Forward (5*′*- 3*′*) | CCCTCGTTTTGCCATGAACC | |
|  | Reverse (5*′*- 3*′*) | GTTGCTGCTGGTTGGAGTTG | |
| CTGF(Human) | Forward (5*′*- 3*′*) | GTTTGGCCCAGACCCAACTA | |
|  | Reverse (5*′*- 3*′*) | GGCTCTGCTTCTCTAGCCTG | |
| CYR61(Human) | Forward (5*′*- 3*′*) | CAGGACTGTGAAGATGCGGT | |
|  | Reverse (5*′*- 3*′*) | GCCTGTAGAAGGGAAACGCT | |
| BIRC5(Human) | Forward (5*′*- 3*′*) | | TGAGAACGAGCCAGACTTGG |
|  | Reverse (5*′*- 3*′*) | | TTTCCTTTGCATGGGGTCGT |
| IGFBP4(Human) | Forward (5*′*- 3*′*) | | CTGAGACTGGCACTTAGCCC |
|  | Reverse (5*′*- 3*′*) | | GGAGTGTCTCCACATGCCAA |
| BMP4(Human) | Forward (5*′*- 3*′*) | | CGGAAGCTAGGTGAGTGTGG |
|  | Reverse (5*′*- 3*′*) | | TCGAGATAGCTTGGACGGGA |
| ALDH1A1(Human) | Forward (5*′*- 3*′*) | | ATCAAAGAAGCTGCCGGGAA |
|  | Reverse (5*′*- 3*′*) | | GCATTGTCCAAGTCGGCATC |
| si-HLF | 5’-GCTGGGCAAATGCAAGAACAT-3’ | | |
| si-YAP1 | 5’- GGCCCUUUGAUUUAGUAUA -3’ | | |
