# Supplementary material for: HLF promotes ovarian cancer progression and chemoresistance via regulating Hippo signaling pathway
Source: Cell Death Dis. 2023 Sep 14;14(9):606. doi: 10.1038/s41419-023-06076-5 (PMC10502110; doi:10.1038/s41419-023-06076-5)
Supplement: Supplementary file 2 — author-contribution-form [file 41419_2023_6076_MOESM2_ESM.pdf]

DECLARATION OF CONTRIBUTIONS TO ARTICLE

ADMC

Manuscript Number: CDDIS-23-1585R

Journal Name: *Cell Death & Disease* (the 'Journal')

Proposed Title of the Contribution:

HLF promotes ovarian cancer progression and chemoresistance via regulating Hippo signaling pathway (the 'Contribution)

Author(s):

Tao Han, Tingsong Chen, Lujun Chen, Kexin Li, Daimin Xiang, Lei Dou, Hengyu Li and Yubei Gu (the 'Authors')

For all *CDDis* articles, each person named as an author in the published version must be able to show he or she has contributed substantially to the article.

Authorship credit should be based on 1) substantial contributions to conception and design, acquisition of data, or analysis and interpretation of data; 2) drafting the article or revising it critically for important intellectual content; and 3) final approval of the version to be published. Authors should meet conditions 1, 2 and 3.

Any person who cannot be shown to have made a substantial contribution to the article cannot be listed as an author in the final version. The name of any person who is deemed to have made a minor contribution can, however, appear in the Acknowledgments section of the article.

Please complete the table below to indicate the contributions of all named authors to the manuscript.

Author Full Name:	Specification of Contribution to the Manuscript:
Tao Han	performed most experiments and statistical analyses
Tingsong Chen	performed part of experiments and statistical analyses
Lujun Chen	performed part of experiments and statistical analyses
Kexin Li	performed part of experiments and statistical analyses
Daimin Xiang	provided support with the experimental techniques
Lei Dou	conceived, designed and supervised the study
Hengyu Li	conceived, designed and supervised the study
Yubei Gu	conceived, designed and supervised the study

Please complete the table below to indicate the contributions of all named authors to the figures.

Figure 1:

Tao Han, Tingsong Chen and Lujun Chen performed Real-time PCR and immunohistochemistry staining.

Figure 2:

Tao Han, Tingsong Chen and Lujun Chen performed flow cytometry, CCK8, clone and sphere formation, Edu, in vitro limiting dilution, transwell and invasion assay. Lujun Chen performed the in vivo animal experiment and immunohistochemistry staining.

Figure 3:

Tao Han performed Real-time PCR and immunohistochemistry staining. Lujun Chen and Kexin Li performed immunofluorescence staining, luciferase reporter and ChIP assay.

Figure 4:

Tao Han, Tingsong Chen and Lujun Chen performed CCK8, sphere formation, Edu, in vitro limiting dilution and invasion assay. Lujun Chen and Kexin Li performed the in vivo animal experiment.

Figure 5:

Tao Han and Tingsong Chen performed Western blot, Real-time PCR, sphere formation and immunohistochemistry staining. Lujun Chen performed Edu, in vitro limiting dilution and invasion assay.

Figure 6:

Tao Han, Tingsong Chen and Lujun Chen performed Western blot, CCK8, clone formation and flow cytometry assay. Lujun Chen and Kexin Li performed the in vivo animal experiment.

Figure 7：
Tao Han, Tingsong Chen and Lujun Chen performed Western blot clone formation and flow cytometry assay. Lujun Chen and Kexin Li performed the in vivo animal experiment.

Signed for and on behalf of the Author(s):	Print Name:	Date:
Yubei Gu	Yubei Gu	8.5.2023
