# Supplementary material for: HLF promotes ovarian cancer progression and chemoresistance via regulating Hippo signaling pathway
Source: Cell Death Dis. 2023 Sep 14;14(9):606. doi: 10.1038/s41419-023-06076-5 (PMC10502110; doi:10.1038/s41419-023-06076-5)

Fig. 3

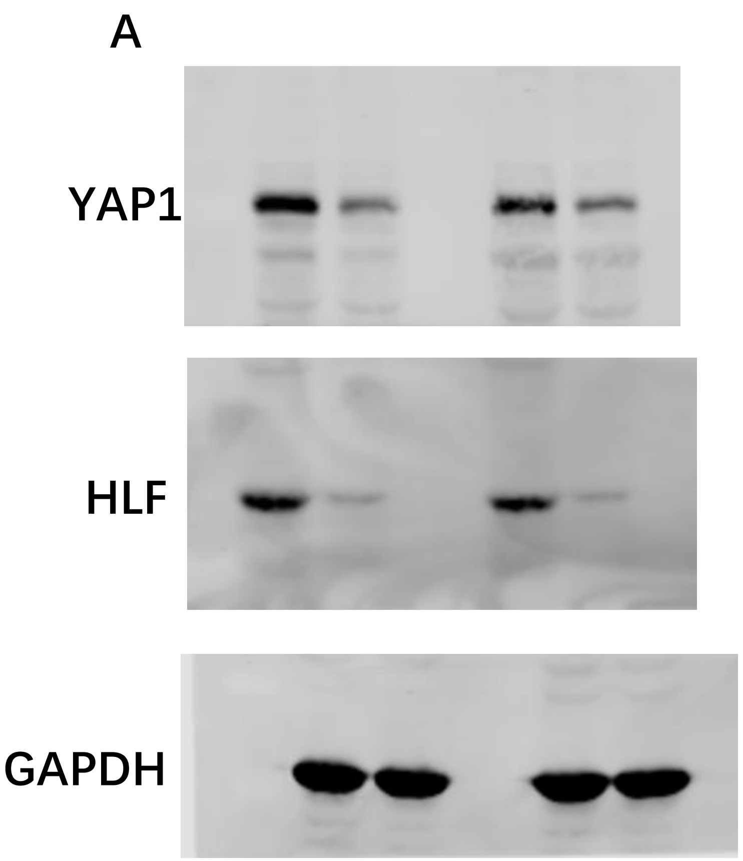

Fig. 5

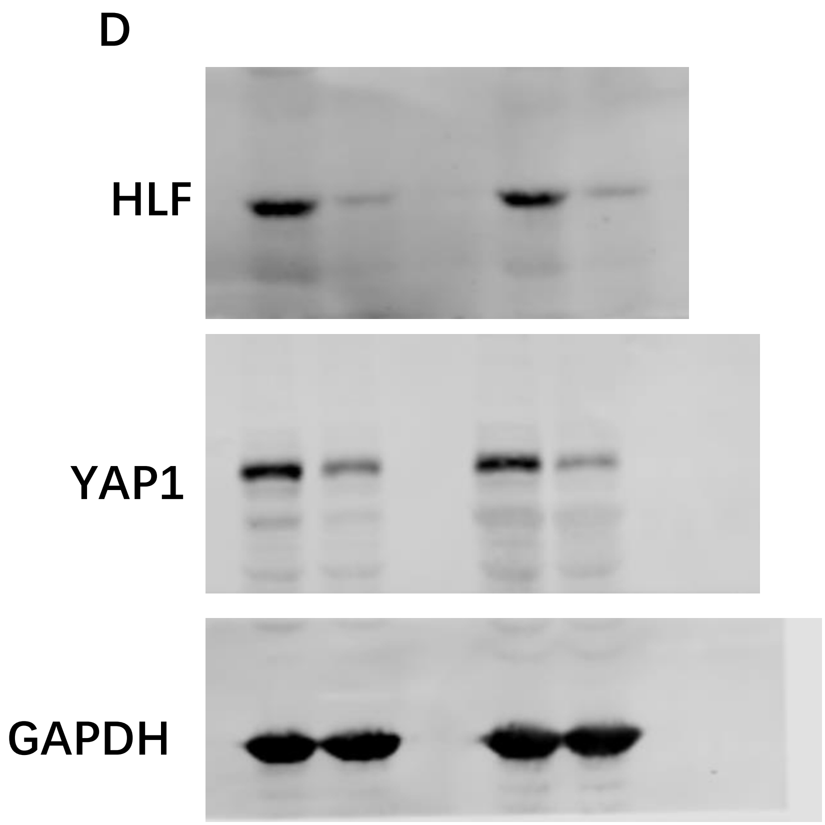

Fig. 6

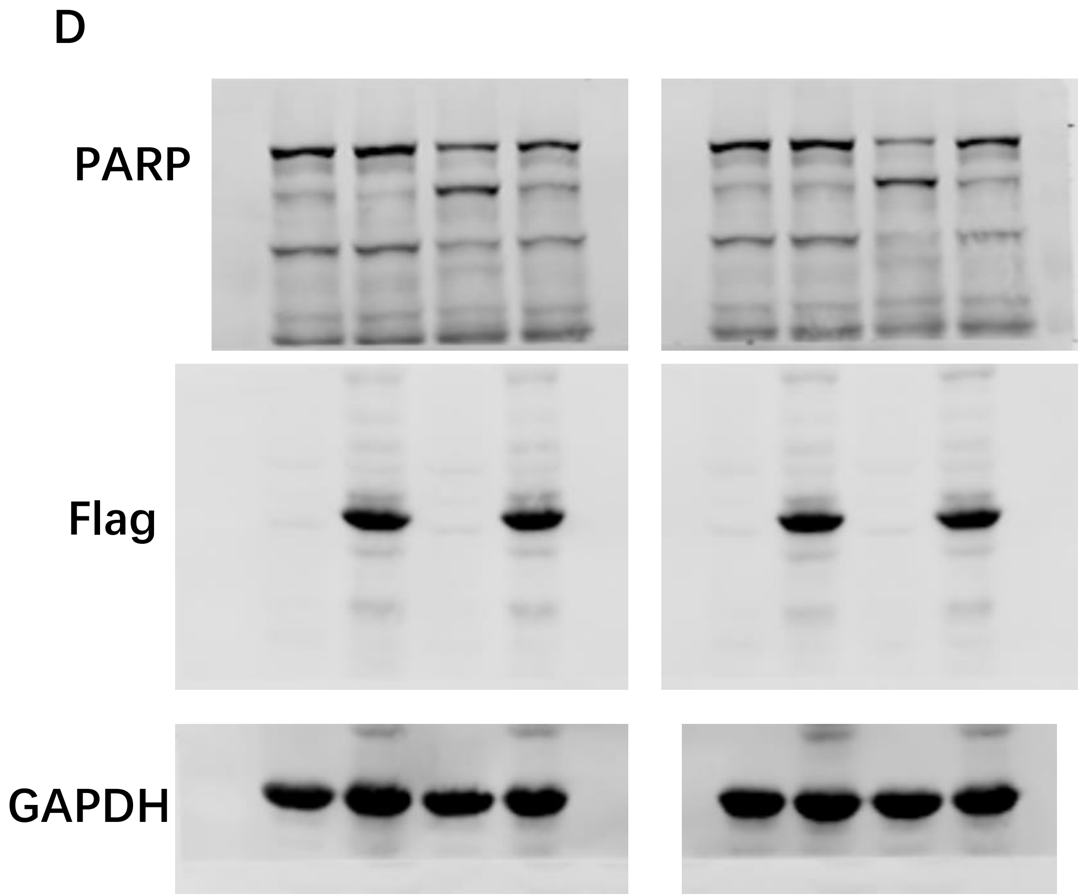

Fig. 7

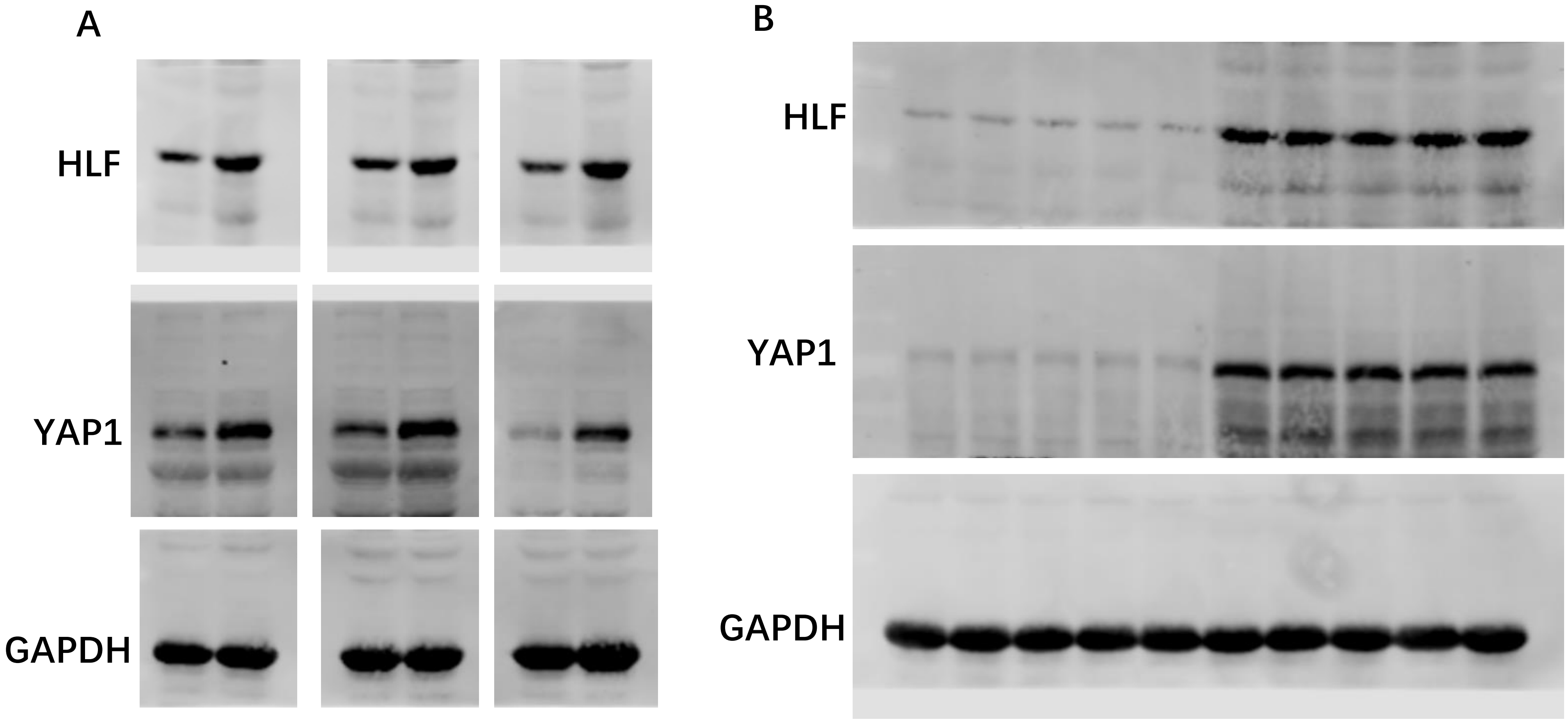

Supplementary Fig. S1

B

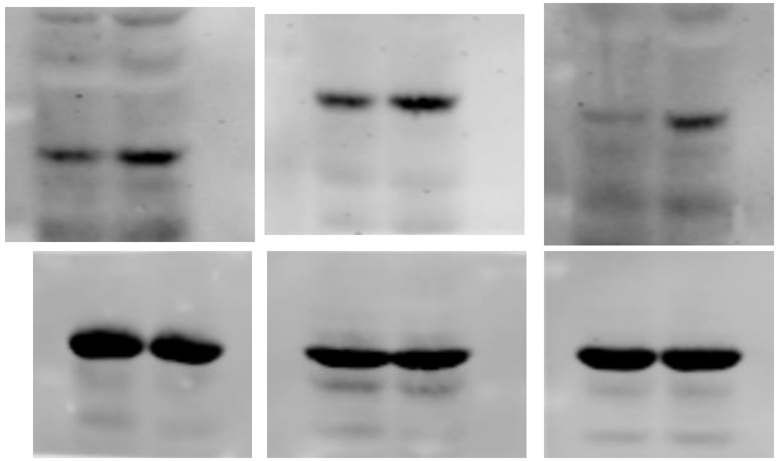

D

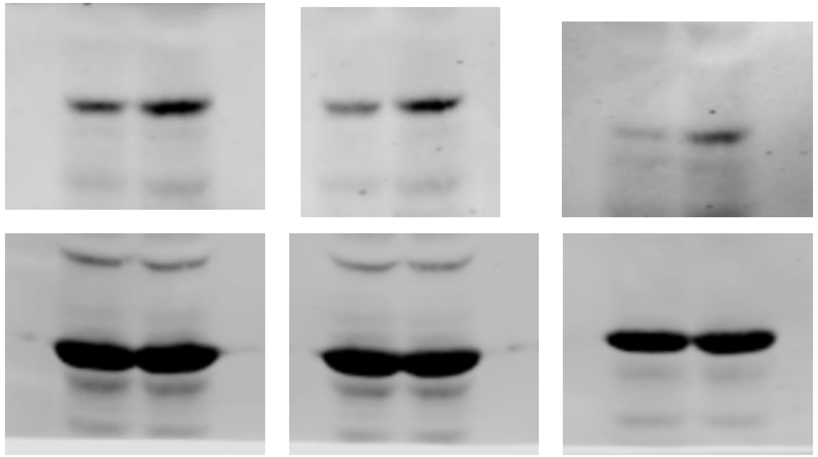

E

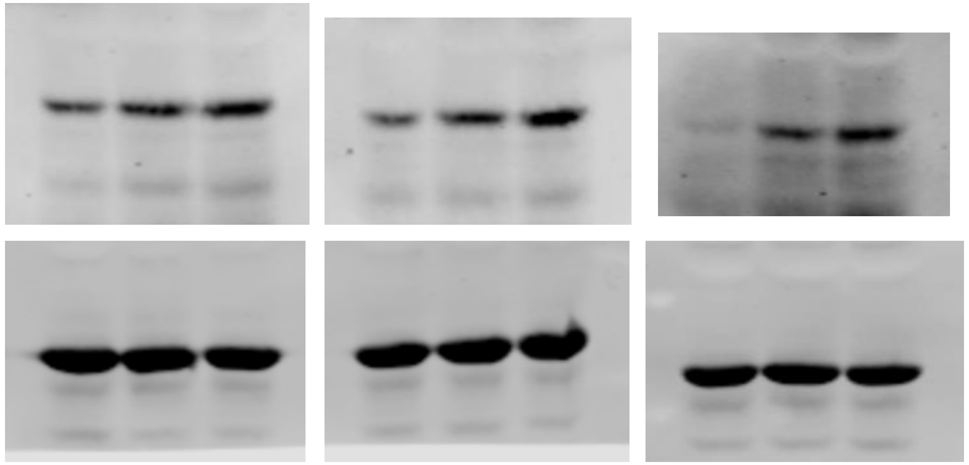

F

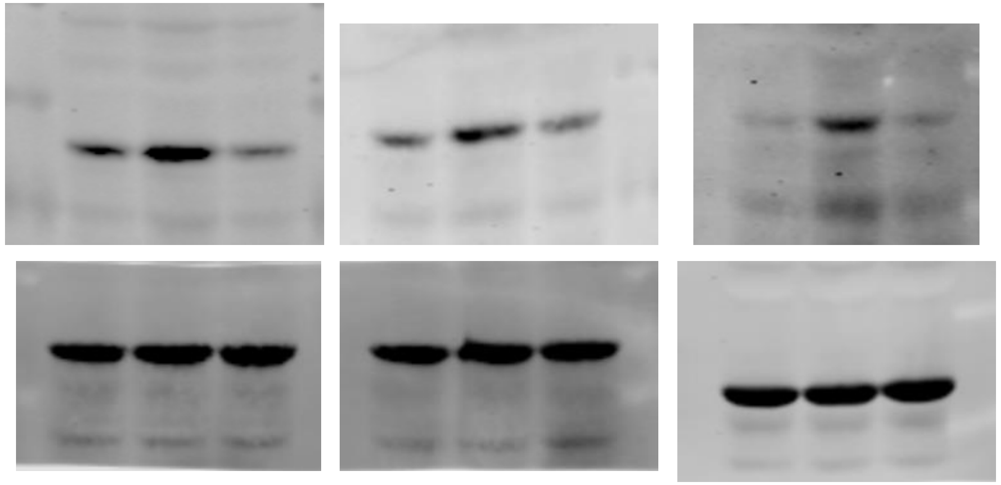

Supplementary Fig. S2

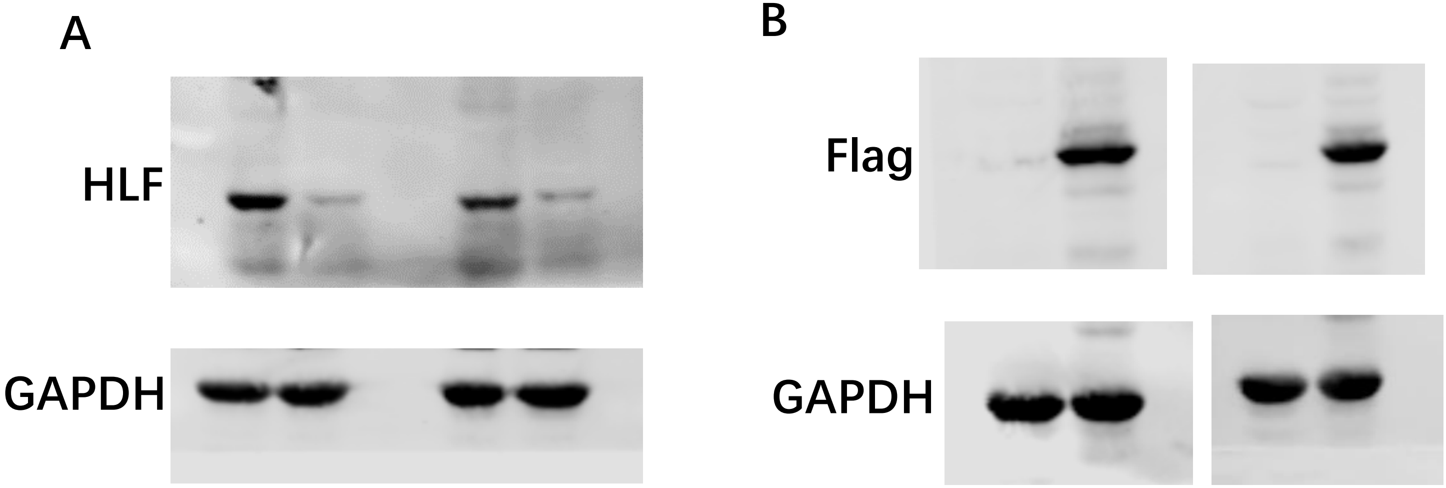

Supplementary Fig. S3

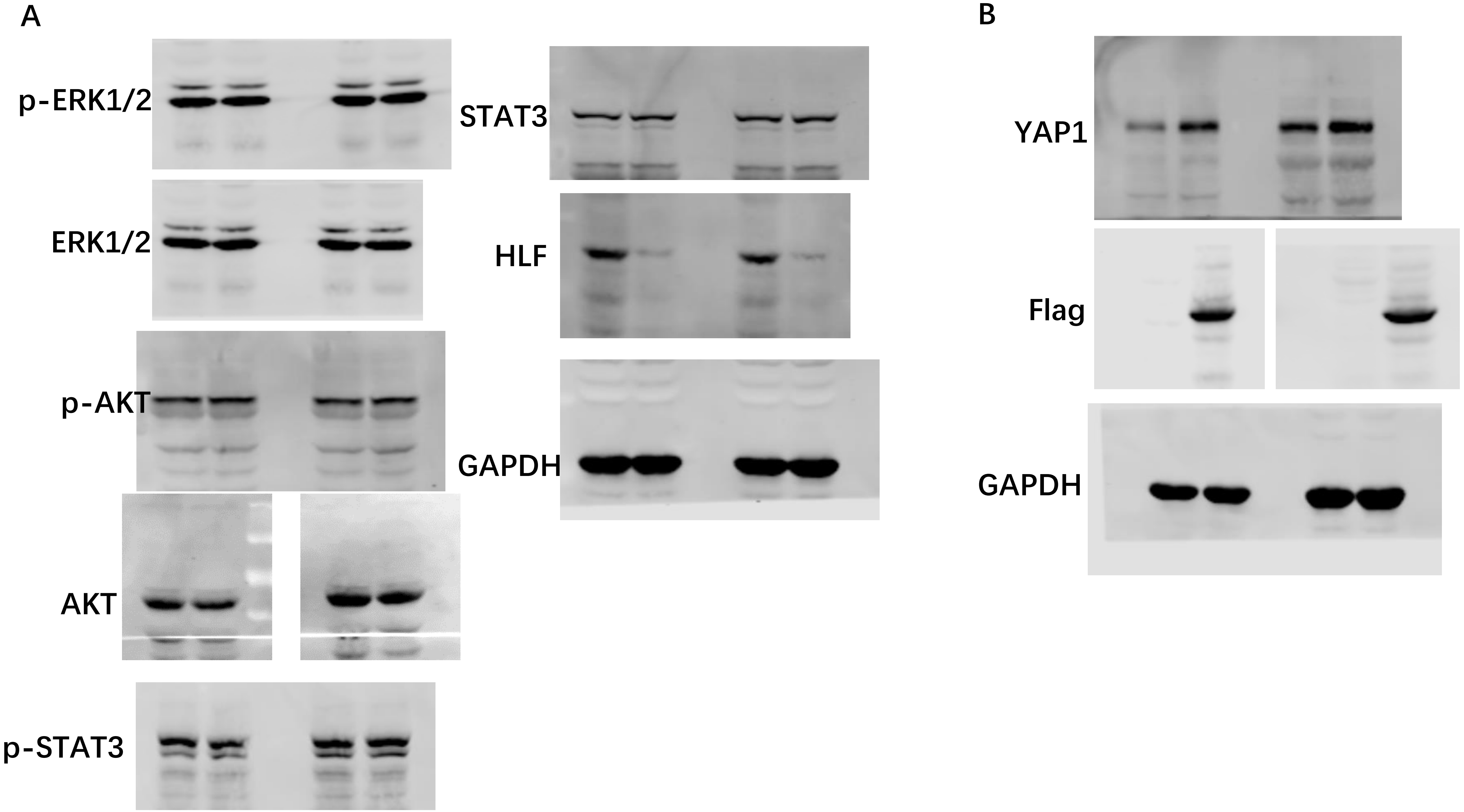

Supplementary Fig. S5

A

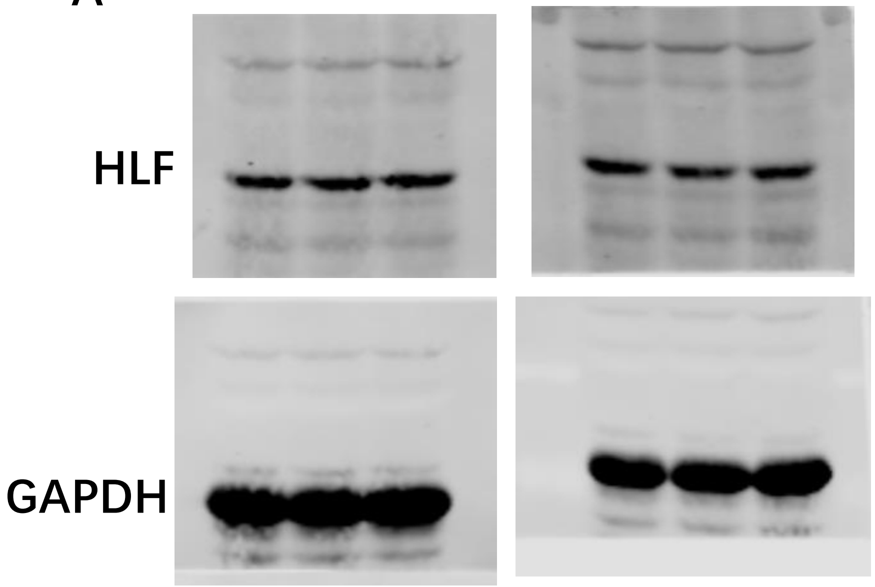

B

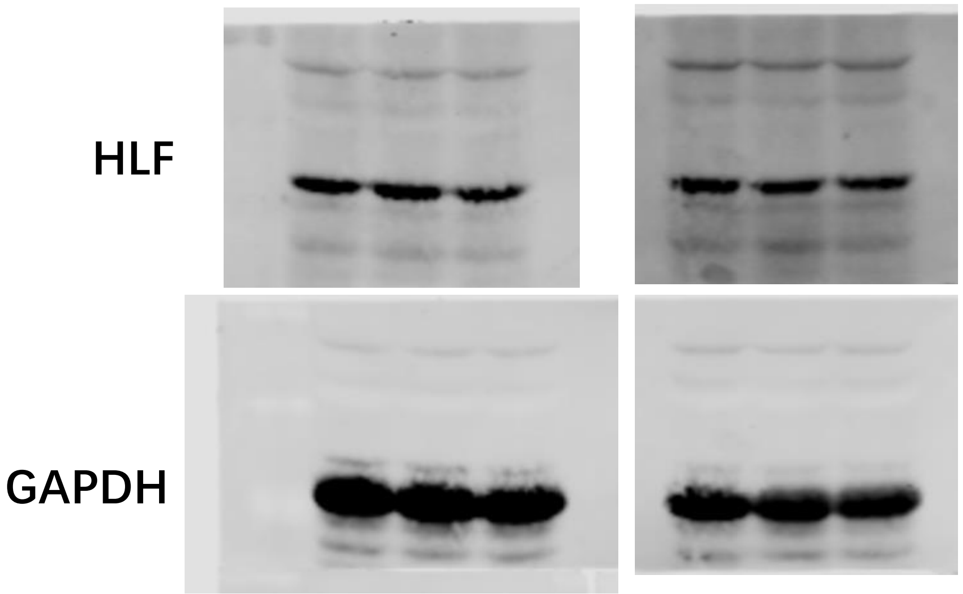

F

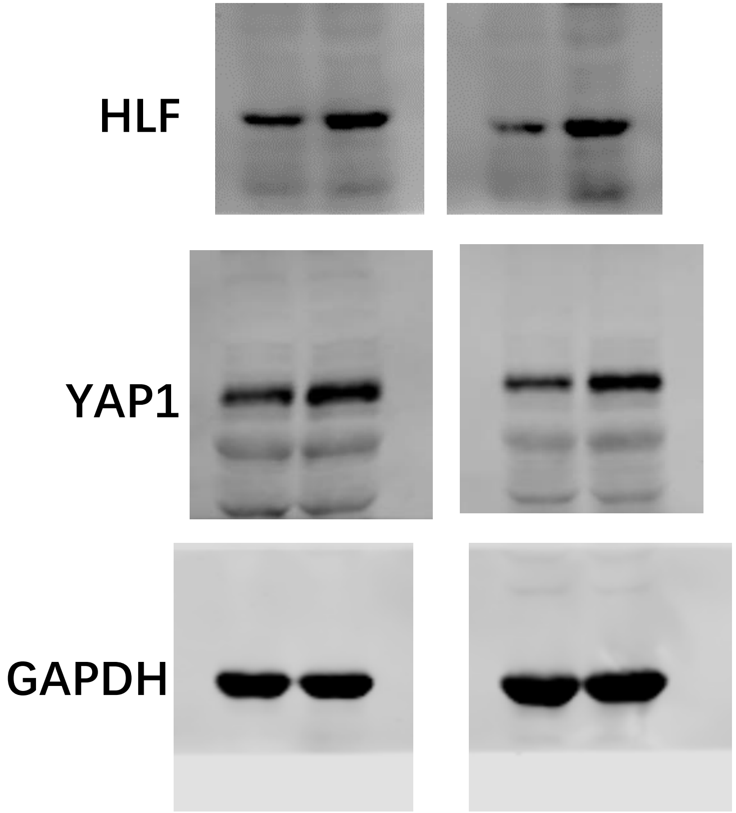

Supplement: Supplementary file 3 — Original Data File [file 41419_2023_6076_MOESM3_ESM.pdf]
